# The KLF16/MYC feedback loop is a therapeutic target in bladder cancer

**DOI:** 10.1186/s13046-024-03224-3

**Published:** 2024-11-18

**Authors:** Lisi Zheng, Jingxuan Wang, Shan Han, Li Zhong, Zefu Liu, Bin Li, Ruhua Zhang, Liwen Zhou, Xianchong Zheng, Zhenhua Liu, Cuiling Zeng, Ruonan Li, Yezi Zou, Liqin Wang, Yuanzhong Wu, Tiebang Kang

**Affiliations:** 1 Sun Yat-Sen University Cancer Center, State Key Laboratory of Oncology in South China, Guangdong Provincial Clinical Research Center for Cancer, 651 Dongfeng Road East, Guangzhou, 510060 People’s Republic of China; 2 Center of Digestive Disease, Scientific Research Center, The Seventh Affiliated Hospital, Sun Yat-Sen University, Shenzhen, 518107 People’s Republic of China; 3 Department of Urology, Xiangya Hospital, Central South University, Changsha, 410008 People’s Republic of China; 4 MOE Key Laboratory of Gene Function and Regulation, State Key Laboratory of Biocontrol, School of Life Sciences, Sun Yat-Sen University, Guangzhou, 510275 People’s Republic of China

**Keywords:** KLF16, MYC, Bladder cancer, BET inhibitors, Chemotherapy sensitivity

## Abstract

**Background:**

Bladder cancer (BLCA) is a common malignancy characterized by dysregulated transcription and a lack of effective therapeutic targets. In this study, we aimed to identify and evaluate novel targets with clinical potential essential for tumor growth in BLCA.

**Methods:**

CRISPR-Cas9 screening was used to identify transcription factors essential for bladder cancer cell viability. The biological functions of KLF16 in bladder cancer were investigated both in vitro and in vivo. The regulatory mechanism between KLF16 and MYC was elucidated through a series of analyses, including RNA sequencing, quantitative polymerase chain reaction (qPCR), RNA immunoprecipitation, Western blotting, Mass spectrometry, Dual-luciferase reporter assays, Cleavage Under Targets and Tagmentation (CUT&Tag) sequencing, OptoDroplets assays, and RNA stability assay. The clinical relevance of KLF16 and MYC in bladder cancer was evaluated through analyses of public databases and immunohistochemistry.

**Results:**

Krüppel-like factor 16 (KLF16) was essential for BLCA cell viability. Elevated expression of KLF16 was observed in bladder cancer tissues, and higher expression levels of KLF16 were correlated with poor progression-free survival (PFS) and cancer-specific survival (CSS) probabilities in BLCA patients. Mechanistically, KLF16 mRNA competed with the mRNA of dual-specificity phosphatase 16 (DUSP16) for binding to the RNA-binding protein, WW domain binding protein 11 (WBP11), resulting in destabilization of the DUSP16 mRNA. This, in turn, led to activation of ERK1/2, which stabilized the MYC protein. Furthermore, KLF16 interacted with MYC to form nuclear condensates, thereby enhancing MYC’s transcriptional activity. Additionally, MYC transcriptionally upregulated KLF16, creating a positive feedback loop between KLF16 and MYC that amplified their oncogenic functions. Targeting this loop with bromodomain inhibitors, such as OTX015 and ABBV-744, suppressed the transcription of both KLF16 and MYC, resulting in reduced BLCA cell viability and tumor growth, as well as increased sensitivity to chemotherapy.

**Conclusions:**

Our study revealed the crucial role of the KLF16/MYC regulatory axis in modulating tumor growth and chemotherapy sensitivity in BLCA, suggesting that combining bromodomain inhibitors, such as OTX015 or ABBV-744, with DDP or gemcitabine could be a promising therapeutic intervention for BLCA patients.

**Supplementary Information:**

The online version contains supplementary material available at 10.1186/s13046-024-03224-3.

## Background

Bladder cancer (BLCA) is the tenth most common cancer worldwide, causing significant morbidity and mortality. The relapsing nature of BLCA, coupled with the necessity for ongoing cystoscopic monitoring, renders it among the most costly cancers to manage [1]. Advances in understanding the genetic landscape of BLCA have led to the identification of actionable therapeutic targets, such as TP53 [2], FGFR3 [3], and ARID1A [4]. Although targeting strategies for these genes have been explored in animal models and early clinical trials, their limited clinical efficacy due to low response rates and resistance remains a challenge [5]. Cisplatin (DDP)-based combination chemotherapies, including the gemcitabine-cisplatin regimen, remain the primary treatment for patients with locally advanced or metastatic bladder cancer. However, the median overall survival has remained at about 15 months for decades, and nearly half of the patients experience recurrence or progression of the disease [6, 7]. Therefore, there is an urgent need to identify and evaluate novel targets with clinical potential.

c-Myc (hereafter MYC) is a multifunctional transcription factor that orchestrates various transcriptional programs involved in cell growth, proliferation, apoptosis, metabolism, and other critical processes under normal conditions [8]. Maintaining MYC at homeostatic levels is crucial for normal cell function. However, pathologically activated or elevated levels of MYC protein contribute to the development of many human cancers, including BLCA [9, 10]. The activity and stability of MYC are primarily regulated through phosphorylation and ubiquitination processes. Within the N-terminal transactivation domain (TAD) of MYC, there are two critical phosphorylation sites: serine 62 (S62) and threonine 58 (T58). Extracellular signal-regulated kinases (ERK) or cyclin-dependent kinases (CDKs) phosphorylate S62, leading to MYC stabilization and functional activation. Subsequent phosphorylation at T58 by glycogen synthase kinase 3 beta targets MYC for recognition and degradation by E3 ubiquitin ligases [11, 12]. In addition, the regulation of MYC activity and stability is also tightly controlled by other proteins in a context-dependent manner. For instance, Bromodomain-containing 4 (BRD4) phosphorylates MYC at T58, leading to MYC ubiquitination and degradation [13]. In acute lymphoblastic leukemia, Aurora B kinase promotes leukemogenesis by directly phosphorylating MYC at serine 67, thereby enhancing its protein stability [14]. DNA polymerase delta 1 promotes the proliferation and metastasis of BLCA by stabilizing MYC via competitively binding with the E3 ubiquitin ligase FBXW7, resulting in decreased ubiquitination-mediated degradation of MYC [15]. Transcriptional abnormalities represent a prominent feature of bladder cancer cells. However, the specific transcription factors that regulate MYC activity and stability in BLCA remain to be elucidated.

The Krüppel-like factor 16 (KLF16) is a DNA-binding transcription factor that regulates a variety of target genes involved in cell proliferation, migration, and apoptosis [16]. Despite emerging studies suggesting a critical role for KLF16 in cancers, the underlying mechanisms and potential therapeutic targeting strategies are not well understood. Here, we demonstrate that *KLF16* is essential for BLCA tumor growth and forms a reinforcing regulatory loop with MYC. Targeting this loop with bromodomain and extraterminal (BET) inhibitors, by impairing the transcription of *KLF16* and *MYC*, leads to reduced cell viability, tumor growth, and increased sensitivity to chemotherapy in BLCA.

## Results

### KLF16 promotes tumor growth in BLCA

**Fig. 1:**
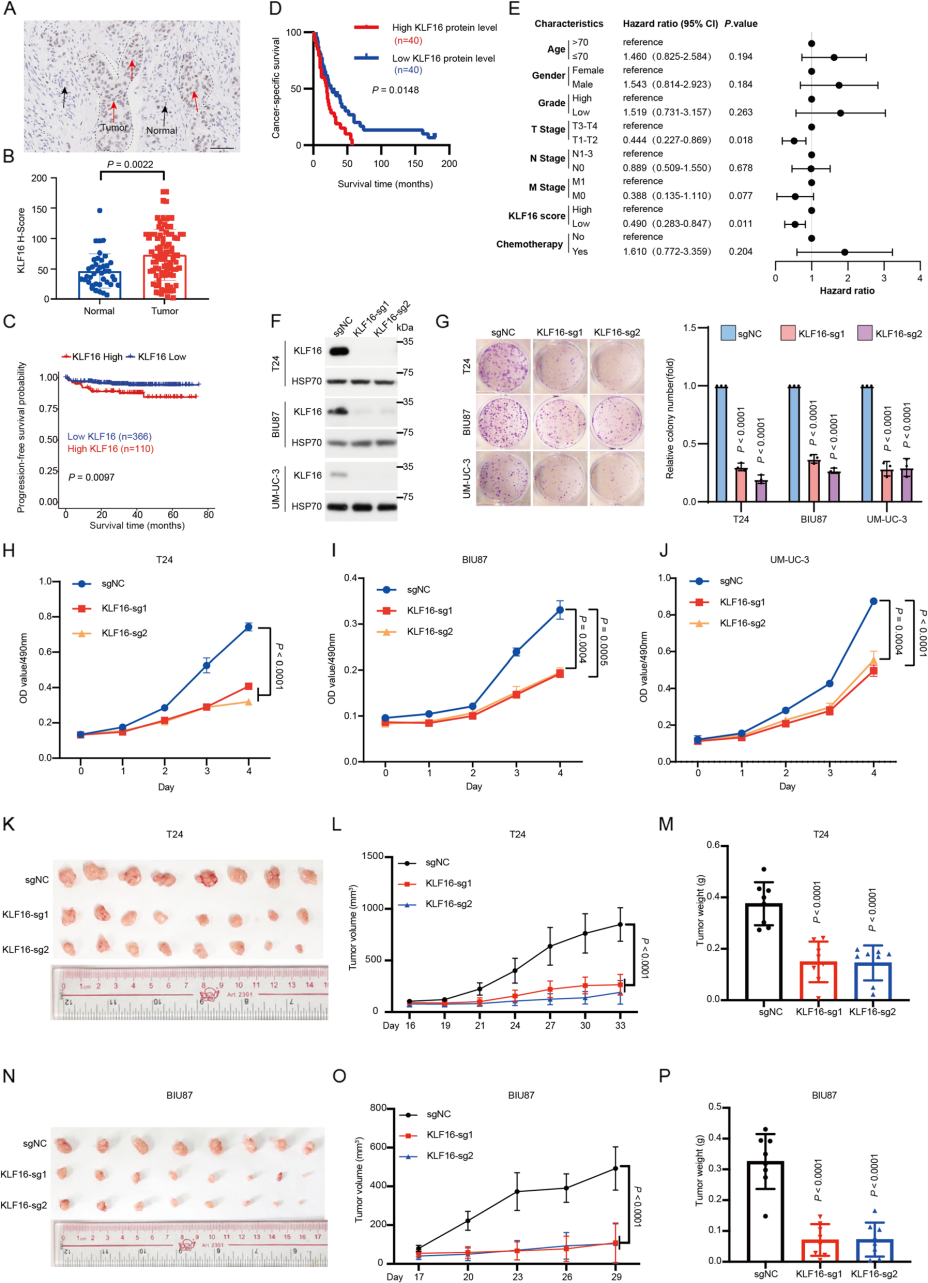
KLF16 promotes tumor growth in BLCA. **A** Representative immunohistochemical images of KLF16 in the human BLCA tissue microarray. Scale bar, 100 μm. **B** Beeswarm plot graph of KLF16 expression in BLCA and adjacent noncancerous tissues used in (**A**). **C** Progression-free survival curves were generated based on the mRNA levels of *KLF16* in BLCA tissues. Data from R2 database: Genomics Analysis and Visualization Platform (http://r2.amc.nl). *P* value was determined by Kaplan–Meier method and compared with the log-rank test. **D** Cancer-specific survival curves were generated based on the protein levels of KLF16 in the 80 paraffin-embedded BLCA tissues. The* P* value was determined by Kaplan–Meier method and compared with the log-rank test. **E** Forest plot of multivariate Cox analysis in BLCA tissues used in (**D**). **F** Western blotting of the indicated proteins in T24, BIU87 and UM-UC-3 cells expressing KLF16-targeted sgRNAs. **G** Representative images (left) and quantification (right) of colonies formed by the indicated BLCA cells used in (**F**). The colony numbers were quantified using ImageJ software. *n* = 3 biologically independent experiments. **H-J** Cell viability of the indicated stable cells in (**F**) was measured by MTT assay at the indicated time points.* n* = 3 biologically independent experiments. **K**-**P** T24 and BIU87 cells expressing KLF16-targeted sgRNAs were subcutaneously injected into nude mice. Representative images of subcutaneous xenograft tumors were shown (**K** and** N**). Tumor volumes were measured at the indicated time points (**L** and **O**). Tumor weight was measured at the end point (**M** and **P**). *n* = 8 nude mice per group. All error bars represent mean ± SD. *P* values in **B**, **G-J**, **M** and **P** were calculated using two-tailed unpaired Student’s t-tests. *P* values in **C**-**E** was determined by the log-rank test. *P* values in **L** and **O** were calculated by two-way ANOVA with Tukey’s multiple comparisons test

To systematically identify transcription factors required for the survival of BLCA cells, we conducted a CRISPR knockout screening using a single guide RNA (sgRNA) library targeting all human transcription factors in bladder cancer cell lines (T24, 5637, and SW780) [17]. Among the candidate genes, we focused on KLF16 (Supplementary Fig. S1A), as its role in human cancers remains poorly explored. Notably, KLF16 was the sole member of the KLF family that significantly impacted cell proliferation (Supplementary Fig. S1B). In addition, immunohistochemistry (IHC) analysis using a tissue microarray (TMA) containing 108 BLCA specimens revealed that KLF16 protein was significantly upregulated in tumor tissues compared to adjacent noncancerous tissues (Fig. 1A, B), and high KLF16 protein levels were associated with worse progression-free survival (PFS) and cancer-specific survival (CSS) probabilities in patients with BLCA (Fig. 1C, D). To identify whether KLF16 could serve as an independent predictive factor from other clinical characteristics, such as stage, grade, and treatment, we employed multivariate Cox regression analysis. As shown in Fig. 1E, the outcomes indicated that KLF16 stood as a significant risk factor for the CSS of patients with BLCA (Hazard ratio = 0.490, 95% *CI*: 0.283–0.847, *P* = 0.011).

To determine the functions of KLF16 in BLCA cells, we knocked out (KO) KLF16 using CRISPR-Cas9 in T24, BIU87, and UM-UC-3 cell lines (Fig. 1F). To further validate the findings and address potential off-target effects of the sgRNAs, we also performed KLF16 knockdown (KD) using shRNA in these cell lines (Supplementary Fig. S1C). Both KO and KD of KLF16 impaired colony formation, cell proliferation, and tumor growth in BLCA cells. (Fig. 1G-P; Supplementary Fig. S1D-N).

### KLF16 depletion attenuates MYC activity in BLCA

**Fig. 2:**
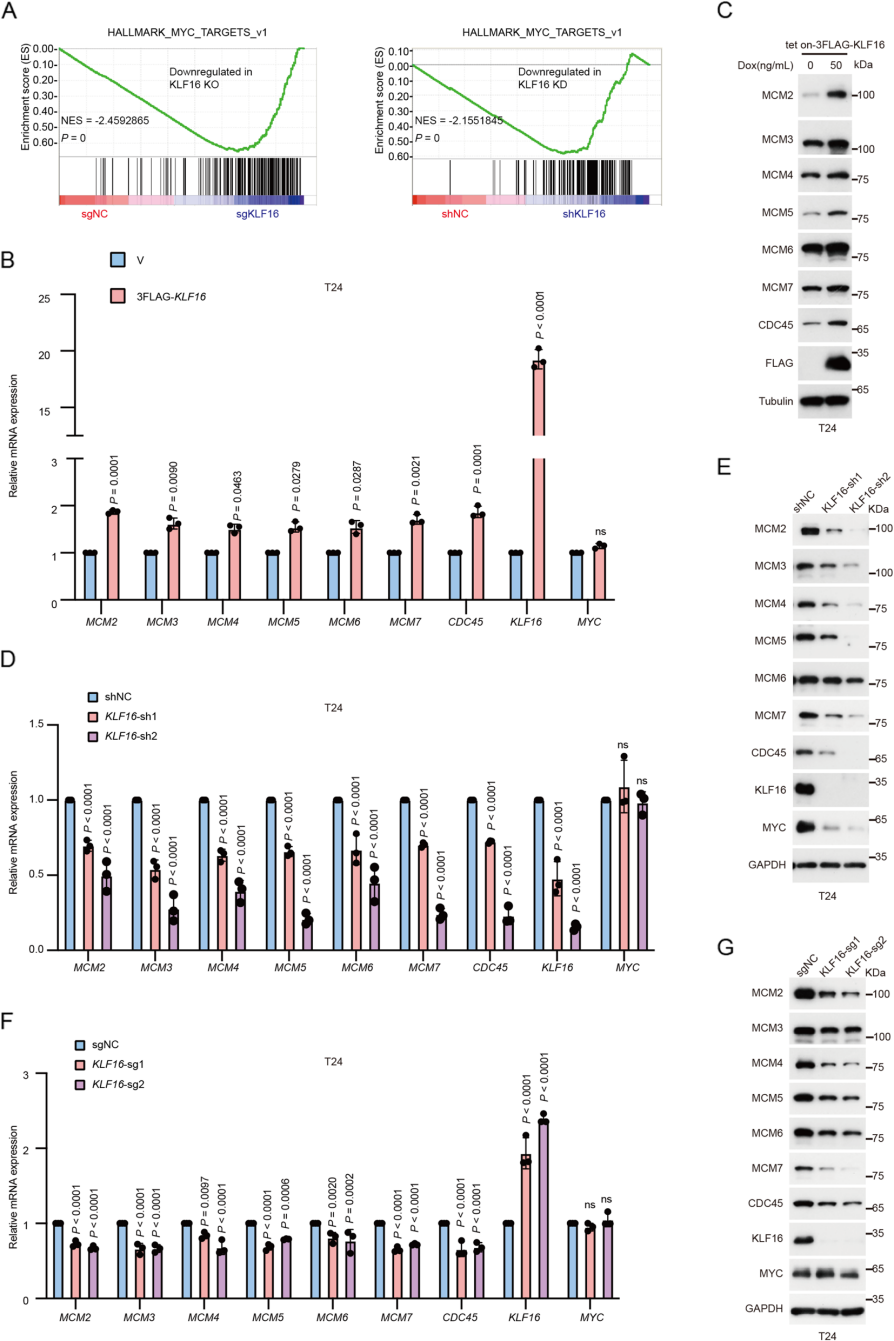
KLF16 depletion attenuates MYC activity in BLCA. **A** GSEA plots evaluating the changes in MYC-targeted gene expression signature upon KLF16 KO (left) or KD (right). NES, normalized enrichment score. KO, knockout; KD, knockdown. **B**, **D**, **F** qPCR analyzing the mRNA levels of *MCM2-7* and *CDC45* in T24 cells stably expressing empty vector (V) or 3FLAG-KLF16 (**B**) and negative control (NC) or shKLF16 (**D**) or sgKLF16 (**F**). ns, not significant.* n* = 3 biologically independent experiments. **C**,** E**,** G** Western blotting assay for the levels of the indicated proteins in the indicated stable cells. All error bars represent mean ± SD and* P* values in **B**, **D**, **F** were calculated using two-tailed unpaired Student’s *t*-tests

To explore how KLF16 regulates cell viability in BLCA, we performed RNA sequencing (RNA-seq) analysis in T24 cells with KLF16 KO and KD. Gene set enrichment analyses (GSEA) revealed a marked downregulation of the MYC-targeted gene expression signature following KLF16 KO or KD (Fig. 2A). When compared with the significantly differentially expressed genes (DEGs) in BLCA from The Cancer Genome Atlas (TCGA) database, we observed that members of replicative CDC45-MCM2-7-GINS (CMG) helicases complex, including minichromosome maintenance protein 2 (MCM2) [18], MCM4 [19], MCM5 [20], MCM6 [21], and cell division cycle protein 45 (CDC45) [22], which are involved in regulating cell proliferation and prognosis in bladder cancers, were enriched (Supplementary Fig. S2A). Quantitative polymerase chain reaction (qPCR) analysis confirmed the upregulation or downregulation of these genes in response to MYC overexpression or depletion (Supplementary Fig. S2B-D). Likewise, these MYC-targeted genes were upregulated and downregulated at both the mRNA and protein levels by overexpression of KLF16 and KLF16 KD or KO in T24 cells, respectively (Fig. 2B-G), indicating that KLF16 may function by regulating MYC, one of the most commonly dysregulated transcription factors in BLCA [23]. Interestingly, KLF16 KD specifically reduced the protein levels of MYC (Fig. 2D-E), while KLF16 KO did not have the same effect (Fig. 2G), implying that *KLF16* mRNA may regulate MYC protein levels in a non-protein-coding dependent manner.

### KLF16 mRNA stabilizes MYC protein via destabilizing DUSP16 mRNA in BLCA

**Fig. 3:**
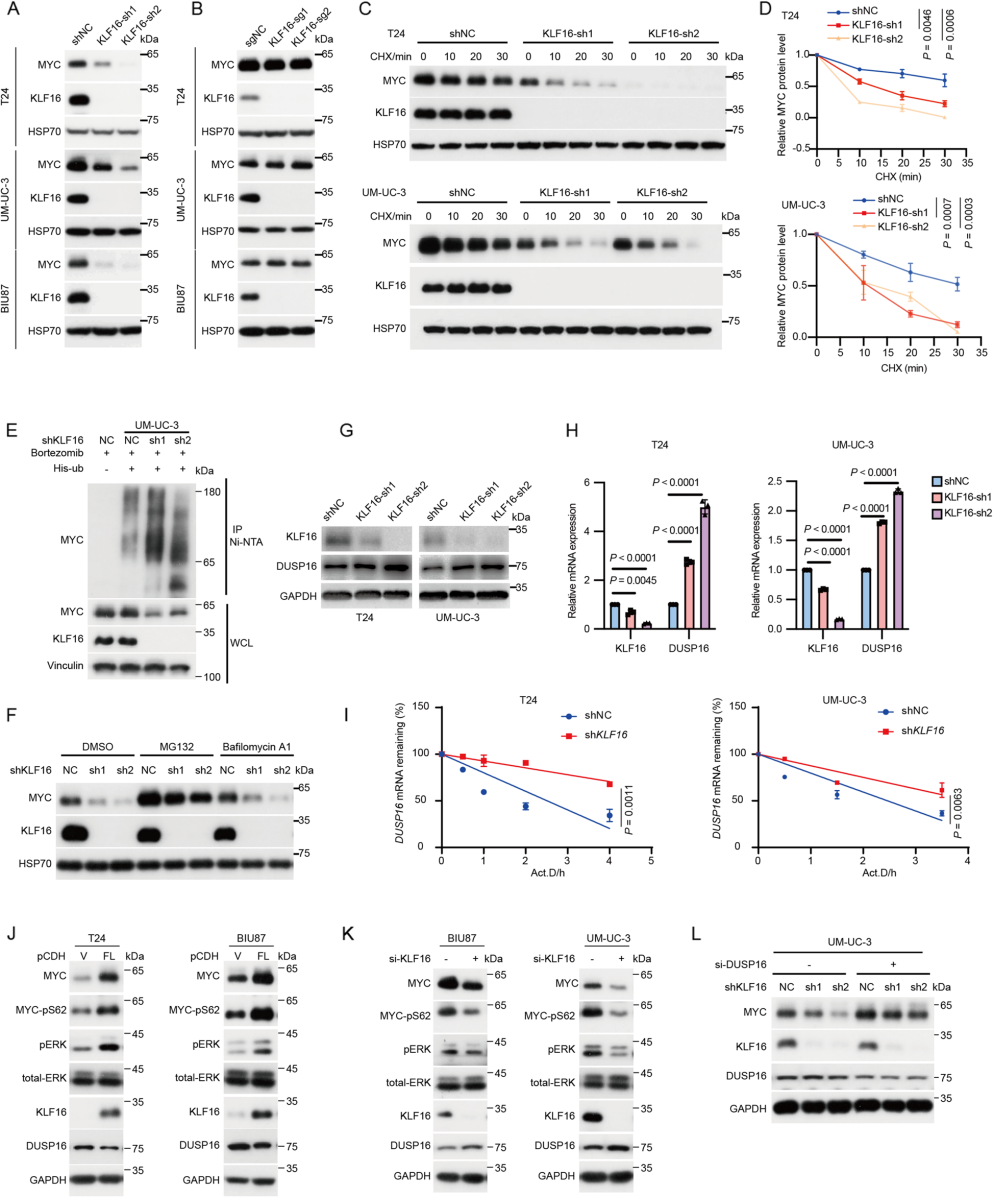
*KLF16* mRNA stabilizes MYC protein via destabilizing *DUSP16* mRNA in BLCA. **A-B** Western blotting assay for the levels of the indicated proteins in the indicated BLCA cells stably expressing KLF16-targeted shRNAs (**A**) or sgRNAs. (**B**)** C-D** T24 and UM-UC-3 cells stably expressing KLF16-targeted shRNAs were treated with 20 μg mL^−1^ cycloheximide (CHX) at the indicated time points and then analyzed by Western blotting (**C**). Quantitation of MYC protein levels was based on the Western blotting results (**D**).* n* = 3 biologically independent experiments. **E** UM-UC-3 cells stably expressing KLF16-targeted shRNAs transfected with His-ub for 24 h were incubated with 1 μM bortezomib for 8 h, and then subjected to IP using Ni-NTA beads followed by Western blotting. Ni-NTA, nitrilotriacetic acid; WCL, whole cell lysate. **F** T24 cells stably expressing KLF16-targeted shRNAs were treated with DMSO, 10 μM MG132 or 1 μM Bafilomycin A1 for 8 h, and then subjected to Western blotting. **G-H** T24 and UM-UC-3 cells stably expressing KLF16-targeted shRNAs were analyzed by Western blotting (**G**) and qPCR (**H**).* n* = 3 biologically independent experiments. **I** The indicated stable cells in (**G**) were treated with 10 μg mL^−1^ actinomycin D (Act.D) at the indicated time points. Total RNA was prepared and *DUSP16* mRNA expression was analyzed by qPCR. The transcript remaining is defined as relative expression at the indicated times compared with the expression level at 0 h. **J** T24 and BIU87 cells stably overexpression of KLF16 full-length mRNA were analyzed by Western blotting. FL, full-length. **K** BIU87 and UM-UC-3 cells transfected with KLF16-targeted siRNAs for 48 h were analyzed by Western blotting. **L** UM-UC-3 cells stably knockdown of KLF16 were transfected with DUSP16-targeted siRNAs for 48 h and then were analyzed by Western blotting. All error bars represent mean ± SD and *P* values were calculated using two-tailed unpaired Student’s *t*-tests

The regulation of MYC protein levels by *KLF16* mRNA, not by KLF16 protein, was further confirmed in other BLCA cells. KLF16 KD significantly reduced MYC protein levels but not mRNA levels (Fig. 3A; Supplementary Fig. S3A), while KLF16 KO had minimal effects on both mRNA and protein levels of MYC (Fig. 3B; Supplementary Fig. S3B). Moreover, KLF16 KD shortened the half-life and increased the ubiquitination of endogenous MYC protein (Fig. 3C-E), whereas ectopic full-length (FL) of *KLF16* mRNA prolonged the half-life and decreased the ubiquitination of endogenous MYC protein (Supplementary Fig. S3C-E). The decrease in MYC protein levels by KLF16 KD was rescued by treatment with the proteasome inhibitor MG132, but not the lysosome inhibitor Bafilomycin A1 (Fig. 3F). These results indicate that *KLF16* mRNA promotes MYC protein stabilization in a non-protein-coding dependent manner.

Next, we sought to explore how *KLF16* mRNA regulates MYC protein stability. Analyzing our RNA-seq data from KLF16 KD cells, we focused on dual-specificity phosphatase 16 (DUSP16) (Supplementary Fig. S3F), as the DUSP family, a subclass of protein tyrosine phosphatases, negatively regulates the ERK activity [24], and ERK is responsible for phosphorylating MYC at serine 62 (MYC-S62), which stabilizes the MYC protein. Indeed, KLF16 KD, but not KLF16 KO, increased both mRNA and protein levels of DUSP16 and prolonged the half-life of *DUSP16* mRNA (Fig. 3G-I; Supplementary Fig. S3G-H). Consistent with this, knockdown and overexpression of wild-type (WT) DUSP16 significantly enhanced and inhibited MYC-S62 phosphorylation, leading to increased and decreased MYC protein levels, respectively (Supplementary Fig. S3I-K), In contrast, overexpression of the DUSP16-C244S mutant [25] (specifically mutated in the phosphatase catalytic domain), had minimal effects on MYC (Supplementary Fig. S3K). Consistently, overexpression and knockdown of *KLF16* mRNA led to increased and reduced MYC-S62 phosphorylation, respectively, as well as altered MYC protein levels without affecting MYC mRNA levels (Fig. 3J, K; Supplementary Fig. S3L-M). More importantly, knockdown of DUSP16 by siRNAs partially rescued the impaired MYC levels caused by *KLF16* KD (Fig. 3L). In contrast, inhibition of ERK1/2 using the specific inhibitor SCH772984, reduced MYC protein stability in KLF16-overexpressing cells (Supplementary Fig. S4A-B). Collectively, these results indicate that *KLF16* mRNA negatively regulates the stability of *DUSP16* mRNA, thereby sustaining ERK activity and promoting MYC protein stabilization.

### KLF16 mRNA negatively regulates DUSP16 mRNA stability by competitively binding to WBP11

**Fig. 4:**
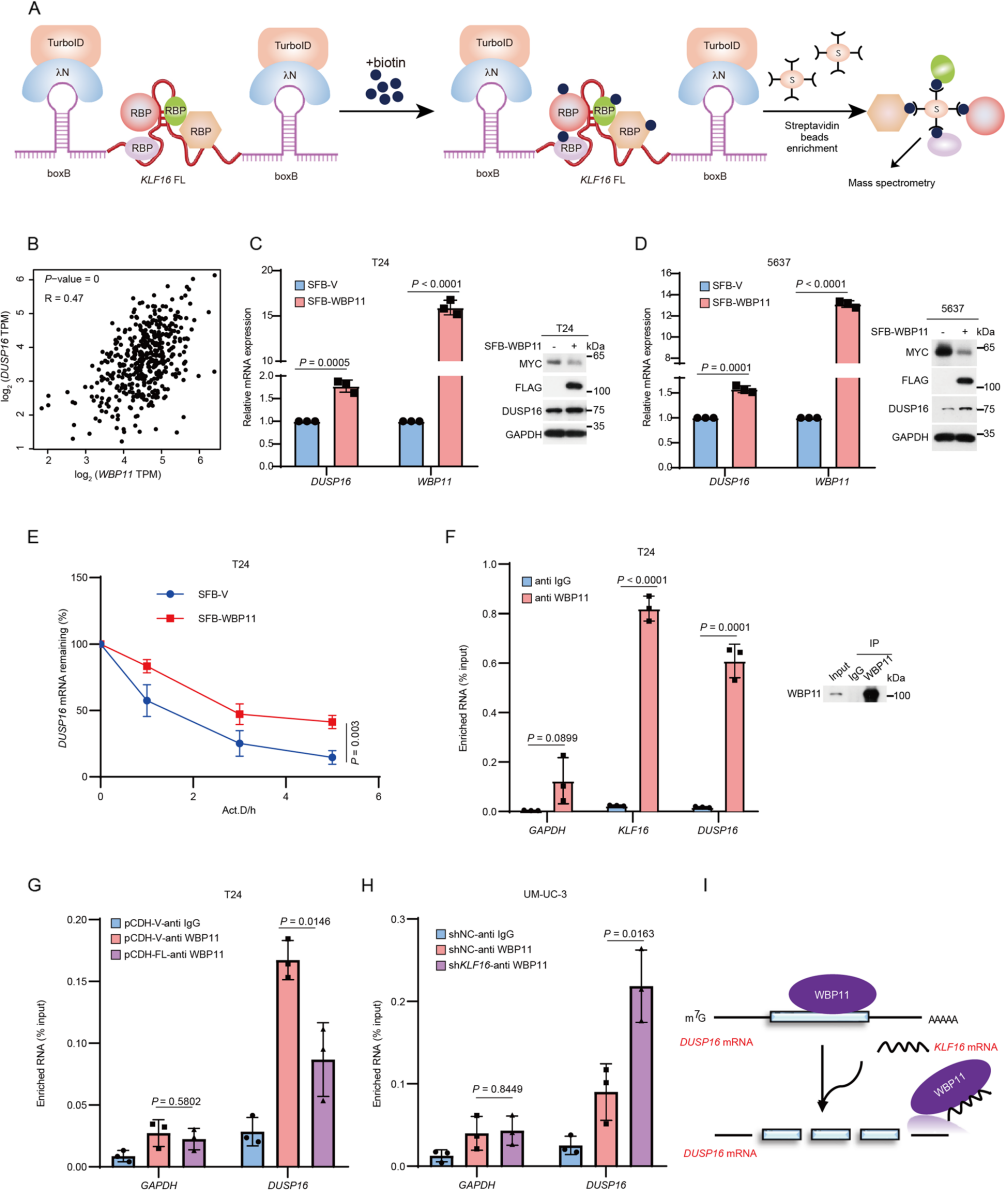
*KLF16* mRNA negatively regulates *DUSP16* mRNA stability by competitively binding to WBP11. **A** Schematics of the RNA–protein interaction detection system. boxB RNA stem-loops (purple) flank KLF16 full-length mRNA (red). λN-HA-TurboID fusion protein binding to boxB sites leads to biotinylation of proteins proximal to inserted KLF16 mRNA sequence in living cells grown in biotin-containing media. Streptavidin (S) beads capture biotinylated protein for mass spectrometry. RBP, RNA binding protein. **B** The scatter plot shows the Pearson correlation of *WBP11* and *DUSP16* mRNA expression in bladder tissues. Data from Gene Expression Profiling Interactive Analysis database (GEPIA): http://gepia.cancer-pku.cn/index.html. *P* value was determined by the log-rank test and the *R*-value was analyzed using Spearman's correlation test. *R*, Spearman correlation coefficient. **C-D** T24 (**C**) and 5637 (**D**) cells stably overexpression of SFB-WBP11 were analyzed by qPCR (left) and Western blotting (right). **E** T24 cells stably overexpression of SFB-WBP11 were treated with 10 μg mL^−1^ actinomycin D (Act.D) at the indicated time points. Total RNA was prepared and *DUSP16* mRNA expression was analyzed by qPCR. The transcript remaining was defined as relative expression at the indicated times compared with the expression level at 0 h. *n* = 3 biologically independent experiments. **F** RIP assay with WBP11 antibody and IgG was performed using T24 lysates and subsequently subjected to qPCR analysis for the indicated RNAs (left). IP enrichment was determined with WBP11 antibody and IgG (right). **G-H** T24 cells stably overexpression of KLF16 FL mRNA (**G**) or UM-UC-3 cells stably knockdown of KLF16 (**H**) were harvested for RIP assay with WBP11 antibody and IgG. Binding of WBP11 to the indicated RNA targets was determined by qPCR. *n* = 3 biologically independent experiments. **I** Proposed model of *KLF16* mRNA destabilizes *DUSP16* mRNA via competitively binding with WBP11. All error bars represent mean ± SD and *P* values were calculated using two-tailed unpaired Student’s *t*-tests unless noted otherwise

We further investigated how *KLF16* mRNA negatively regulates *DUSP16* mRNA stability. Given that long non-coding RNAs (lncRNAs), generally considered non-protein-coding transcripts, participate in mRNA stability through interaction with RNA-binding proteins [26], we hypothesized that *KLF16* mRNA might regulate the stability of *DUSP16* mRNA through such a mechanism. To investigate the proteins associated with *KLF16* mRNA, we performed an optimized proximity labeling assay coupled with mass spectrometry (Fig. 4A) [27, 28]. Among the proteins identified by mass spectrometry (Supplementary Material 4), the RNA-binding protein WW domain binding protein 11 (WBP11) aroused our interest due to its strong positive correlation with *DUSP16* in BLCA (Fig. 4B). Indeed, ectopic expression of SFB-WBP11 increased both mRNA and protein levels of DUSP16, extended the half-life of *DUSP16* mRNA, and decreased MYC protein levels (Fig. 4C-E). RNA immunoprecipitation (RIP) assays confirmed the association of WBP11 with both *DUSP16* and *KLF16* mRNA (Fig. 4F), raising the possibility that *KLF16* mRNA may reduce the half-life of *DUSP16* mRNA by interfering with the binding of WBP11 to *DUSP16* mRNA. This was the case, as overexpression of *KLF16* mRNA and *KLF16* KD led to a decreased and increased interaction between WBP11 and *DUSP16* mRNA, respectively (Fig. 4G, H). Taken together, these results suggest that *KLF16* mRNA disrupts the interaction between WBP11 and *DUSP16* mRNA, thereby destabilizing *DUSP16* mRNA (Fig. 4I).

### KLF16 and MYC colocalize on chromatin, and KLF16 facilitates MYC binding to target gene promoters

**Fig. 5:**
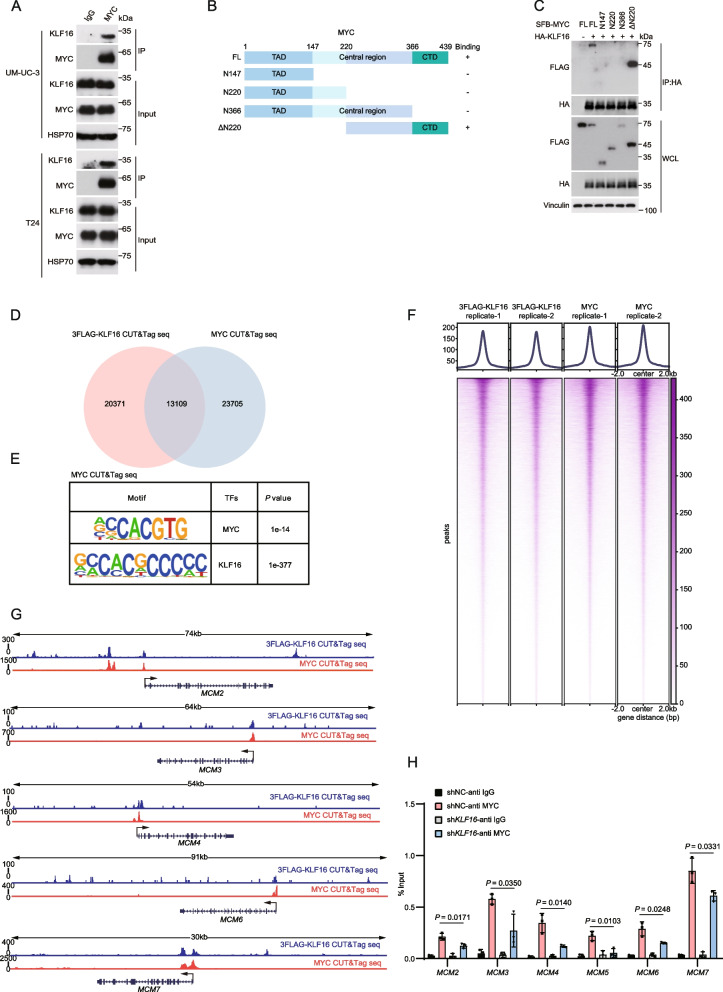
KLF16 and MYC colocalize on chromatin, and KLF16 facilitates MYC binding to target gene promoters. **A** Endogenous KLF16 was immunoprecipitated with endogenous MYC in UM-UC-3 and T24 cells. IP was performed with anti-IgG or anti-MYC antibody. IP, immunoprecipitation. **B** Schematic of SFB-MYC truncations used for co-IP assays in (**C**). **C** HEK293T cells were co-transfected with the indicated plasmids and then were analyzed by immunoprecipitation using anti-HA beads followed by Western blotting. **D-E** Venn diagram (**D**) and HOMER motif analysis (**E**) of 3FLAG-KLF16 and MYC CUT&Tag sequencing binding regions. **F** Heat map of the binding pattern of 3FLAG-KLF16 and MYC. kb, kilobases; FDR, false discovery rate. **G** Track view of 3FLAG-KLF16 and MYC CUT&Tag sequencing density profile on *MCM2, MCM3, MCM4, MCM6 and MCM7* genomic regions in T24 cells displayed by Integrative Genomics Viewer (IGV) software. **H** ChIP-qPCR analysis of MYC occupancy at the indicated gene promoter regions in T24 cells stably expressing sh*KLF16* using IgG or anti-MYC antibody. All error bars represent mean ± SD and *P* values in (**H**) were calculated using two-tailed unpaired Student’s *t*-tests

Since both KLF16 KO and KD repressed MYC-target genes (Fig. 2), and in addition to the stabilization of MYC protein by *KLF16* mRNA as shown above, KLF16 protein may also regulate the transcriptional activity of MYC. Given that KLF16 binds to GC and GT boxes [29], which are similar to the MYC binding motif (E-box sequences, CACGTG), we hypothesized that KLF16 and MYC may co-bind genomic regions to activate target genes. To test this hypothesis, we first validated the interaction between KLF16 and MYC at their endogenous levels by co-immunoprecipitation in UM-UC-3 and T24 cells (Fig. 5A). Moreover, we generated several truncated mutants of MYC based on its conserved functional domains [30], and found that the C-terminal domain (CTD) of MYC is responsible for its interaction with KLF16 (Fig. 5B, C). Second, Cleavage Under Targets and Tagmentation (CUT&Tag) assays revealed that KLF16 and MYC form an extensive regulatory partnership (Fig. 5D). More than 13,000 genomic regions are commonly targeted by these two proteins (39.2% of KLF16-binding sites and 35.6% of MYC-binding sites). In addition, canonical MYC and KLF16 motifs were enriched at these regions (Fig. 5E), suggesting co-occupancy by KLF16 and MYC. This observation was further supported by the highly similar distribution patterns of MYC and KLF16 binding sites across chromatin (Fig. 5F). More importantly, both CUT&Tag sequencing analysis in T24 cells, and ChIP-seq analysis in multiple cell lines from the Encyclopedia of DNA Elements (ENCODE) project [31, 32] showed that MYC and KLF16 peaks were enriched at the promoter regions of the *MCM2-7* complex (Fig. 5G; Supplementary Fig. S5A), and *KLF16* KD significantly reduced MYC occupancy at *MCM2-7* gene promoters (Fig. 5H). Collectively, these results indicate that KLF16 and MYC colocalize on chromatin, and that KLF16 promotes MYC binding to the promoters of target genes.

### KLF16 forms nuclear condensates with MYC, thereby enhancing the MYC’s transcriptional activity in BLCA

**Fig. 6:**
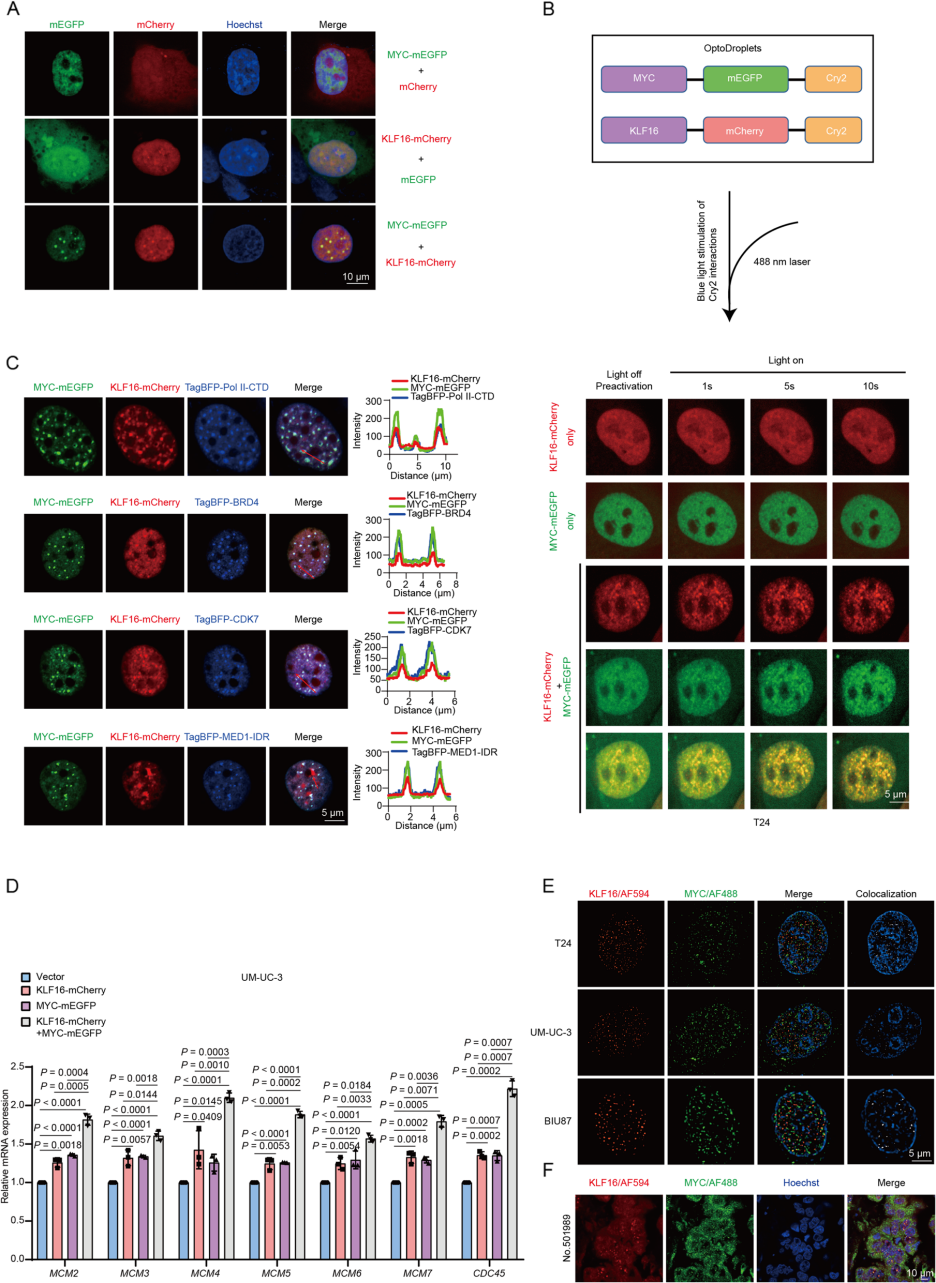
KLF16 forms nuclear condensates with MYC, thereby enhancing the MYC’s transcriptional activity in BLCA. **A** Condensate formation was analyzed in T24 cells transfected with the indicated plasmids. **B** T24 cells were transfected with KLF16-mCherry-Cry2 and MYC-mEGFP-Cry2 separately or together. Images were collected after illumination by a 488 nm laser at the indicated times. **C** Confocal microscopy images of representative condensates in T24 cells co-transfected with the indicated plasmids and stained with the indicated antibodies (left). The corresponding line scan analyses are plotted on the right. **D** qPCR analyzing the mRNA levels of *MCM2-7* and *CDC45* in UM-UC-3 cells transient expressing KLF16-mCherry and MYC-mEGFP separately or together. *n* = 3 biologically independent experiments. **E** SIM analysis of endogenous KLF16 and MYC localization in the indicated cells with anti-KLF16 and anti-MYC antibody. Hoechst 33,342 (prepared in PBS) was used to stain nuclei. **F** Representative immunofluorescence images of nuclear condensates analyzed in tissue samples from bladder patients by co-stained with anti-KLF16 and anti-MYC as indicated. Patient numbers were shown. The experiments in **A**, **B**, **C**, **E** were repeated three times with similar results. All error bars represent mean ± SD and *P* values were calculated using two-tailed unpaired Student’s *t*-tests

To explore how KLF16 and MYC colocalize on chromatin to influence target genes, we hypothesized that KLF16 and MYC may form condensates to activate target genes, as multiple regions of the amino acid sequences of KLF16 and MYC were scored as intrinsically disordered regions (IDRs) using IUPred2 [33] (Supplementary Fig. S6A, B). Previous work has demonstrated that transcription factors, such as MYC, P53, OCT4, etc*.*, can form condensates to enrich the transcription apparatus components and maximize gene expression [34]. Indeed, KLF16-mCherry was able to form nuclear condensates with MYC-monomeric enhanced green fluorescent protein (MYC-mEGFP) (Fig. 6A). Using the optoDroplet assay [35], blue light induction enhanced condensate formation between KLF16-mCherry and MYC-mEGFP when fused with Cry2 (Fig. 6B; Supplementary Fig. S6C). Notably, within these KLF16/MYC condensates, multiple transcriptional machinery components, including RNA polymerase II C-terminal domain (Pol II-CTD), BRD4, CDK7, and MED1-IDR, were enriched (Fig. 6C). Cotransfection of KLF16-mCherry with MYC-mEGFP further increased mRNA levels of MYC-targeted genes, including *MCM2-7* and *CDC45*, compared to transfection with either construct alone (Fig. 6D). Additionally, using a Gal4-responsive reporter system, we also found that cotransfection of the N-terminal domain of KLF16 (Gal4-KLF16-N) could significantly enhance MYC activity (Supplementary Fig. S6D). More importantly, small and colocalized condensates formed by endogenous KLF16 and MYC were observed through super-resolution imaging in BLCA cell lines and tumor tissues (Fig. 6E, F). Taken together, these results demonstrate that KLF16 forms nuclear condensates with MYC to enhance MYC’s transcriptional activity in BLCA.

### MYC transcriptionally upregulates KLF16

**Fig. 7:**
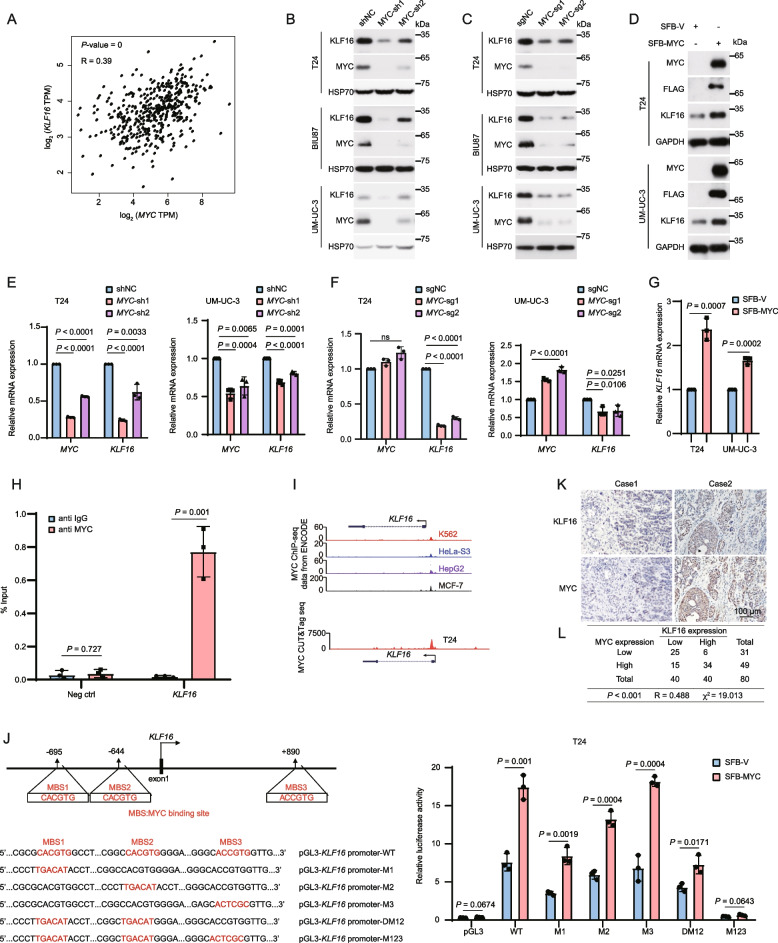
MYC transcriptionally upregulates *KLF16*. **A** The scatter plot shows the Pearson correlation of *MYC* and *KLF16* mRNA expression in BLCA tissues. Data from GEPIA database: http://gepia.cancer-pku.cn/index.html . *P* value was determined by the log-rank test and the *R*-value was analyzed using Spearman's correlation test. **B-G** The BLCA cell lines with MYC depletion or overexpression were analyzed by Western blotting (**B-D**) and qPCR (**E–G**). *n* = 3 biologically independent experiments. **H** ChIP-qPCR analysis of MYC occupancy at *KLF16* promoter region in T24 cells. Neg ctrl, negative control. *n* = 3 biologically independent experiments. **I** Track view of MYC ChIP-seq density profile on KLF16 genomic region in the indicated cell lines from published data sets displayed by UCSC Genome Browser: http://genome.ucsc.edu [36] (upper panel). MYC CUT&Tag seq tracks in gene loci of *KLF16* in T24 cells displayed by IGV software (lower panel). ChIP-seq data from the ENCODE database (https://www.encodeproject.org). **J** Schematic presentation of MYC binding sites on the KLF16 locus (left panel)**.** T24 cells expressing SFB-MYC were transfected with the indicated wild-type (WT) or mutants of KLF16 promoter, along with the Renilla control reporter for 24 h. Then, cells were analyzed for the relative luciferase activity (right panel). MBS, MYC binding site. **K** Representative immunohistochemical images of both KLF16 and MYC from 80 paraffin-embedded BLCA tissues. Scale bar, 100 μm. **L** Crosstab shows the distribution of cancer tissues in the bladder cancer tissues used in (**K**) according to the median H-Score of KLF16 and MYC. The *P* value and chi-square were analyzed using Pearson's chi-squared test, and the *R*-value was analyzed using Spearman's correlation test. All error bars represent mean ± SD and *P* values were calculated using two-tailed unpaired Student’s *t*-tests unless noted otherwise

Interestingly, there was a positive correlation between *KLF16* and *MYC* mRNA levels within the TCGA dataset (Fig. 7A). Given that KLF16 did not influence *MYC* mRNA levels (Fig. 2), we speculated that MYC might transcriptionally regulate KLF16, forming a positive feedback loop. Indeed, deletion and overexpression of MYC significantly suppressed and increased both mRNA and protein levels of KLF16, respectively (Fig. 7B-G), and ChIP-qPCR analysis also confirmed the binding of MYC to the promoter region of *KLF16* in T24 cells (Fig. 7H). Supportively, both ChIP-seq analysis in multiple cell lines from the ENCODE project and CUT&Tag sequencing analysis in T24 cells revealed that MYC bound to the *KLF16* promoter (Fig. 7I). Subsequently, the sequence of the KLF16 promoter was analyzed, and we found two consensus E-box sequences (CACGTG), designated as MYC binding sites 1 and 2 (MBS1/2), along with a similar E-box site (ACCGTG), referred to as MYC binding site 3 (MBS3). A dual-luciferase reporter assay revealed that MYC did bind to MBS1, MBS2, and MBS3 in the *KLF16* promoter, and each motif was functional, as the transcriptional activation of *KLF16* by *MYC* was abolished when all three motifs were mutated (Fig. 7J). These results suggest that MYC transcriptionally upregulates KLF16 to form a positive feedback loop, and such a loop has clinical relevance, as there was a positive correlation between KLF16 and MYC levels assessed by IHC in human bladder cancer tissues (Fig. 7K, L).

### BET inhibitors inhibit tumor growth and enhance chemotherapy sensitivity in BLCA

**Fig. 8:**
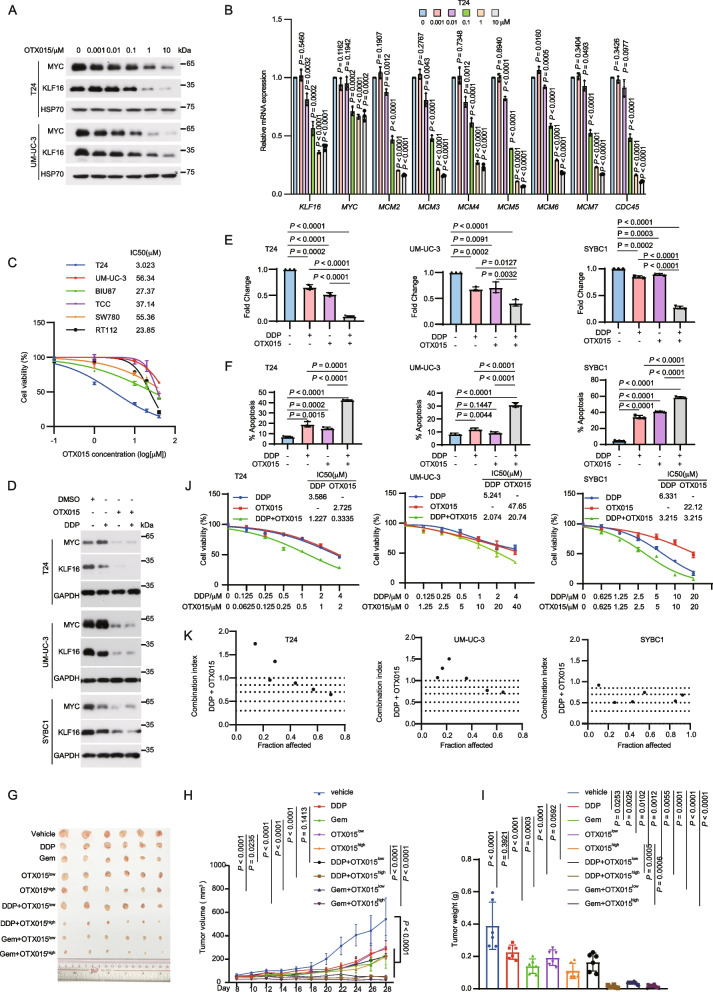
OTX015 suppresses tumor growth and enhances chemotherapy sensitivity in BLCA. **A** T24 and UM-UC-3 cells were treated with increasing concentrations of OTX015 for 48 h and then were analyzed by Western blotting. **B** T24 cells in (**A**) were subjected to qPCR analysis for the indicated mRNA levels. **C** The indicated bladder cancer cell lines were treated with the indicated concentration of OTX015 (0, 0.1 μM, 1 μM, 10 μM, 20 μM, 40 μM) for 48 h, and then the relative cell growth rates were determined by MTT assay.* n* = 3 biologically independent experiments. **D-F** T24, UM-UC-3 and SYBC1 cells were treated with DMSO, OTX015 (10 μM for UM-UC-3 and SYBC1, 1 μM for T24) or DDP (2.5 μM) separately or together, the indicated proteins were determined by Western blotting (**D**), colony formation (**E**) and flow cytometry analysis of apoptosis (**F**).* n* = 3 biologically independent experiments. **G-I** Mice bearing T24 tumors were randomly divided into the indicated groups (*n* = 6 mice per group). DDP (5 mg kg^−1^) or gemcitabine (Gem, 50 mg kg^−1^) were injected intraperitoneally twice weekly. OTX015 (25 mg kg^−1^ & 50 mg kg^−1^) were daily given via intragastric administration for about 3 weeks. Tumor volumes (**H**) and tumor weights (**I**) were measured. **J** T24, UM-UC-3 and SYBC1 cells were treated with the indicated concentration of OTX015 or DDP alone or in combination for 48 h, and then the relative cell growth rates were determined by MTT assays. *n* = 3 biologically independent experiments. **K** The Combination Index (CI) of OTX015 and DDP was calculated based on results from (**J**) by using CalcuSyn. CI < 1, = 1, and > 1 indicate synergism, additive effect, and antagonism, respectively. All error bars represent mean ± SD. *P* values in **H **were calculated by two-way ANOVA with Tukey’s multiple comparisons test.* P* values in **B**,** E–F**, **I** were calculated using two-tailed unpaired Student’s *t*-tests

Given the critical role of the KLF16/MYC loop in BLCA, drugs that effectively disrupt this loop may benefit BLCA patients. However, targeting MYC directly remains a challenge, partly due to its intrinsically disordered structure. Indirectly inhibiting MYC by targeting proteins involved in its transcriptional regulation has emerged as an effective strategy for treating MYC-dependent tumors. Notably, BET inhibitors have demonstrated promising results in blocking MYC transcription [37, 38]. Consequently, we investigated whether BET inhibitors could effectively disrupt the KLF16/MYC loop. Indeed, the BET bromodomain inhibitor OTX015, currently under evaluation in clinical trials, inhibited *MYC*, *KLF16* and their co-regulated target genes, thereby impairing cell viability in a dose-dependent manner (Fig. 8A-C; Supplementary Fig. S7A). Considering that single-agent therapies for advanced cancers are rarely curative due to the rapid development of drug resistance [39], we hypothesized that a combination therapy involving OTX015 with chemotherapeutic agents, such as DDP and gemcitabine, could be a more effective strategy, as KLF16 KD significantly reduced the half-maximal inhibitory concentration (IC50) of DDP and gemcitabine in BLCA cells (Supplementary Fig. S7B, Fig. S8A). Indeed, as evidenced by colony formation, apoptosis, and xenografts in nude mice, the combination of OTX015 with either DDP or gemcitabine was more effective at reducing colony formation, increasing apoptosis, and impeding tumor growth compared to either agent alone (Fig. 8D-I; Supplementary Fig. S7C-E, Fig. S8B-G). Furthermore, the combined toxicity of the two drugs was evaluated using CalcuSyn [40], which showed that the drugs had a synergistic effect at most concentrations (Fig. 8J, K; Supplementary Fig. S8H, I). Similarly, ABBV-744, another novel BET bromodomain inhibitor, exhibited behavior similar to OTX015 (Supplementary Fig. S9 and S10). These findings suggest that blocking the KLF16/MYC loop with BET inhibitors enhances the sensitivity of BLCA cells to chemotherapy.

## Discussion

The *KLF16* mRNA encodes an evolutionarily conserved transcription factor that functions as either a tumor suppressor or an oncogene in a context-dependent manner [41–43]. In our study, we found that KLF16 is overexpressed and promotes tumor growth in BLCA. However, data from TCGA reveal that the percentage of *KLF16* amplification is less than 4% in BLCA, and the main factors leading to *KLF16* upregulation in BLCA have not been fully elucidated. Our findings reveal a positive feedback loop between KLF16 and MYC, critical for maintaining their high expression levels in BLCA. This reciprocal activation may explain why both KLF16 and MYC are overexpressed in BLCA, even at a low rate of gene amplification for both.

Research has revealed the existence of bifunctional RNAs that possess both coding and non-coding functions. For instance, certain mRNAs exhibit dual roles by encoding proteins while also participating in non-coding regulatory processes [44]. Additionally, lncRNAs can encode small peptides [45]. Another interesting example is the steroid receptor RNA activator (SRA), which serves as an RNA mediator for eukaryotic transcriptional gene activation. Simultaneously, SRA can also encode highly conserved SRA proteins that perform various functions [46]. Furthermore, a recent study identified a novel polypeptide encoded by the long non-coding RNA LINC00961, known as small regulatory polypeptide of amino acid response (SPAR). SPAR localizes to late endosomes/lysosomes and negatively regulates mTORC1 activation by interacting with lysosomal v-ATPase [47]. In addition, the *p53* mRNA can directly bind to the RING domain of the E3 ubiquitin ligase MDM2, blocking the enzyme’s activity and increasing *p53* mRNA translation and protein stability [48]. Here, we found that *KLF16* mRNA serves as a novel bifunctional molecule, regulating MYC protein activity and stability. Mechanistically, the KLF16 protein could enhance MYC activity, while *KLF16* mRNA competes with *DUSP16* mRNA for binding to WBP11, resulting in reduced *DUSP16* mRNA stability. This competition leads to increased levels of pERK1/2/pS62-MYC and subsequent MYC accumulation.

Previous studies have reported that MYC undergoes liquid–liquid phase separation to regulate transcription [49]. However, the mechanism by which KLF16 and MYC colocalize on chromatin to promote transactivation of target genes remains unknown. Our research demonstrates that KLF16 and MYC collaborate to activate target gene expression by forming colocalized condensates. Within these condensates, multiple transcriptional machinery components—including RNA polymerase II C-terminal domain (RNA Pol II- CTD), MED1-IDR, BRD4, and CDK7 are enriched, facilitating the activation of target genes such as *MCM2-7* (Fig. 6C-D). A Gal4-responsive reporter assay also confirmed that the N-terminal domain of KLF16 plays a crucial role in promoting MYC activity (Supplementary Fig. S6D). Since previous studies have revealed that the TAD of mammalian transcription factors is required for condensate formation and transcriptional activity [34], we hypothesize that the TAD of MYC and the N-terminal domain of KLF16 may be indispensable for condensate formation and transactivation, which warrants further investigation in the future.

BET inhibitors have emerged as an effective strategy for blocking MYC transcription by targeting proteins involved in its regulatory network [38]. For example, targeting the FGFR3/MYC loop with either JQ1 or inhibitors of FGFR3, P38 or AKT has shown promising therapeutic potential in BLCA patients with aberrant FGFR3 activation [50]. However, in clinical practice, resistance to anti-FGFR therapies and monotherapy with BET bromodomain inhibitors has been observed [51, 52]. Our study demonstrates that disrupting the KLF16/MYC loop with BET inhibitors, such as OTX015 or ABBV-744, in combination with chemotherapeutic agents like DDP or gemcitabine, significantly inhibits tumor growth and enhances the sensitivity of BLCA cells to chemotherapy. Therefore, the combination of BET inhibitors with DDP or gemcitabine may represent a promising therapeutic intervention for BLCA patients.

## Conclusions

**Fig. 9:**
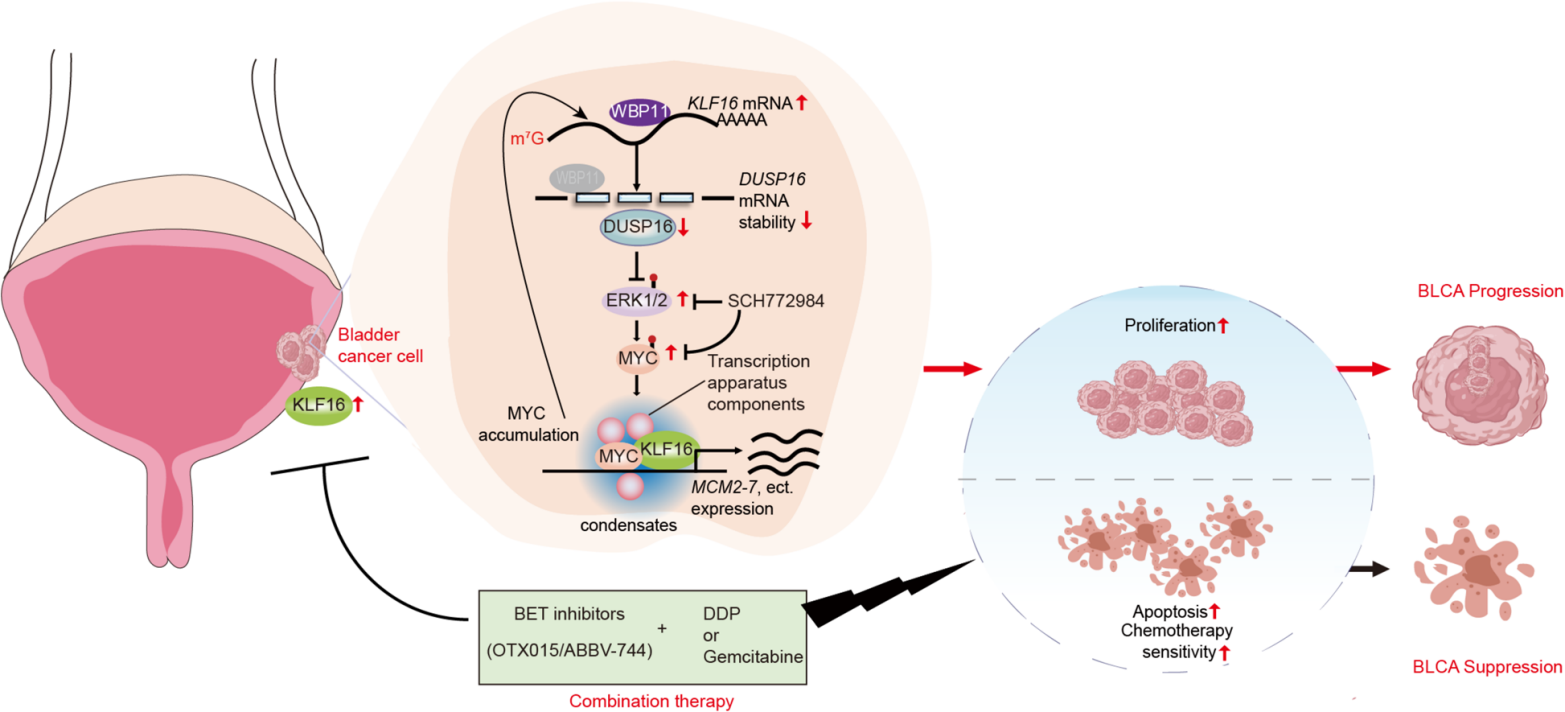
Mechanism schematic of this study. The schematic diagram illustrates the function of KLF16/MYC loop in BLCA. In BLCA, KLF16 is overexpressed. *KLF16* mRNA competes with *DUSP16* mRNA for binding to WBP11, resulting in the reduction of both *DUSP16* mRNA stability and DUSP16 protein levels. This, in turn, activates the ERK1/2 pathway, which stabilizes MYC protein. In contrast, inhibition of ERK1/2 by SCH772984 reduces MYC protein stability. Besides, KLF16 and MYC can form nuclear condensates that recruit transcription apparatus components, accelerating the transcription of target genes, such as *MCM2-7*. Meanwhile, MYC transcriptionally activates *KLF16* by directly binding to the *KLF16* promoter, creating the KLF16/MYC positive feedback loop. Targeting this loop with the BET inhibitors (such as OTX015 and ABBV-744) suppresses *KLF16* and *MYC* transcription, reducing cell viability and tumor growth, as well as increasing sensitivity of BLCA cells to DDP or gemcitabine

In this study, as illustrated in Fig. 9, our research reveals the crucial role of the KLF16/MYC regulatory axis in modulating tumor growth and chemotherapy sensitivity in BLCA, suggesting that combining BET inhibitors, such as OTX015 or ABBV-744, with DDP or gemcitabine could be a promising therapeutic intervention for BLCA patients.

## Materials and methods

### Cell lines and cell culture

Human cell lines T24, UM-UC-3, 5637, BIU87, and human embryonic kidney HEK293T were cultured in Dulbecco's modified Eagle's medium supplemented with 10% fetal bovine serum and 0.5% penicillin/streptomycin at 37 °C with 5% CO_2_. The patient-derived cell line (PDC) SYBC1 was cultured under the same conditions in RPMI 1640 with 10% FBS and 0.5% penicillin/streptomycin. Cell lines T24, UM-UC-3, 5637, and HEK293T were acquired from the American Type Culture Collection (ATCC), whereas the BIU87 cell line was obtained from the Kunming Cell Bank at the Chinese Academy of Sciences. The SYBC1 cell line was generously provided by Dr. Zhuowei Liu of the Sun Yat-sen University Cancer Center. All human cell lines were confirmed to be mycoplasma-negative and authenticated by short tandem repeat (STR) profiling analysis.

### Plasmid construction, virus transduction

Human KLF16, MYC, DUSP16, and WBP11 cDNAs were amplified by PCR and cloned into the pSIN or pcDNA3.1 vectors, with or without tags (3xFlag, SFB, HA, EGFP, mCherry). The full-length mRNA of *KLF16* was cloned into pCDH vectors. Mutants of SFB-DUSP16-C244S were generated from WT SFB-DUSP16 constructs using a one-step cloning kit (Vazyme, C113-02). Constructs for tagBFP-Pol II-CTD, MED1-IDR, BRD4, and CDK7 were obtained as previously described [53]. All shRNAs targeting human KLF16 and MYC were cloned into the PLKO.1 vector. sgRNAs were designed using GUIDES web tool (http://guides.sanjanalab.org/) and cloned into the lentiCRISPR v2 vector. The sequences of all shRNAs and sgRNAs used are provided in Supplementary Material 2.

Lentivirus packaging was performed in HEK293T cells by cotransfecting with the targeted plasmids, psPAX2, and pMD2.G using polyethyleneimine (PEI). After transfection for 6 to 8 h, the cell culture medium was replaced, and the virus-containing medium was collected after an additional 48 h. Transduced cell populations were typically selected 48 h post-infection.

### qPCR

Total RNA was isolated using the RNAprep Pure Cell/Bacteria Kit (TIANGEN) and reverse transcribed with HiScript® II Q RT SuperMix for qPCR (+ gDNA wiper) (Vazyme) according to the manufacturer’s instructions. qPCR was conducted using the ChamQ Universal SYBR qPCR Master Mix (Vazyme) with the LightCycler 480 Instrument II system (Roche). The qPCR primers used are provided in Supplementary Material 2.

### Western blotting and coimmunoprecipitation (Co-IP)

Cells were lysed with RIPA buffer (50 mM Tris–HCl, pH 8.0; 150 mM NaCl; 0.5% NP-40; 5 mM EDTA; protease inhibitor cocktails and phosphatase inhibitors). The supernatant was collected via centrifugation (30 min, 4 °C, 12,000 rpm) and then boiled at 100 °C for 10 min after adding 1 × SDS sample buffer. Proteins were resolved by 8%-12% SDS–PAGE and transferred onto polyvinylidene difluoride membranes (Millipore). The membranes were blocked with 5% nonfat milk (1 h at room temperature) and incubated with the indicated primary antibodies (overnight at 4 °C), followed by incubation with horseradish peroxidase–conjugated secondary antibody (1 h at room temperature). The chemiluminescent signaling was detected by using ECL reagents (Tanon). The antibodies used and their concentrations are listed in Supplementary Material 3.

For co-IP, the clarified cell lysates were incubated with antibody-coated beads or agarose for 2 h or overnight at 4 °C. For endogenous co-IP assay, the clarified cell lysates were first incubated with specific primary antibodies overnight at 4 °C, followed by the addition of protein A/G agarose beads and further incubation for 4 h at 4 °C. Afterwards, the immune-precipitates were washed four times with cold RIPA buffer and then boiled at 100 °C for 10 min after adding 1 × SDS sample buffer.

### Colony formation assay

Stable cells were seeded in six-well plates at a density of 500 cells per well. After 7–10 days, when visible clones had formed, they were washed with phosphate-buffered saline (PBS), fixed in methanol, and stained with 0.1% crystal violet. Colonies with more than 50 cells were quantified using ImageJ software.

### OptoDroplets assay

The optoDroplets assay was performed as described previously [35]. Briefly, T24 and HEK293T cells were transfected with KLF16-mCherry-Cry2 and MYC-mEGFP-Cry2 separately or together. Cells were illuminated with a 488-nm laser, and images were captured for mEGFP, mCherry, or both signals at the indicated time using a spinning disk confocal microscope (Nikon CSU-W1).

### MTT assay

BLCA stable cells were seeded into 96-well plates at a density of 2,000 cells (T24, UM-UC-3, SYBC1 stable cells) or 4,000 cells (BIU87 stable cells) per well and incubated for the indicated time. Subsequently, MTT solution was added to each well at a final concentration of 0.5 mg mL^−1^ followed by incubation for 4 h at 37 °C. The formazan crystals were dissolved in DMSO, and the optical density (OD) was measured at 490 nm using a microplate reader. The IC50 values were calculated using GraphPad software with Nonlinear regression, Dose–response inhibition, log (inhibitor) versus normalized response Variable slope methods.

### Apoptosis assay

BLCA stable cells were seeded into 6-well plates and incubated for 24 h, then treated with the indicated drugs for 24–48 h. Apoptotic cells were collected and subjected to apoptosis assay using the Annexin V-FITC/PI Apoptosis Detection Kit (KGA107, KeyGEN BioTECH) following the manufacturer’s instructions.

### Dual-luciferase reporter assay

Cells were seeded in 24-well plates and incubated for 24 h. They were then cotransfected with 0.5 µg of a promoter-luciferase plasmid and 10 ng of pRL-CMV (Renilla luciferase) using Lipofectamine 3000. After 48 h of transfection, luciferase activity was measured using a Dual-luciferase Reporter Assay kit (Promega) following the manufacturer’s instructions. Renilla luciferase was utilized as an internal control to normalize luminescence levels.

### RNA–protein interaction detection based on TurboID and MS

The following procedures were performed as described previously [27, 28]. To detect proteins interacting with full-length *KLF16* mRNA, we first generated two constructs: a puromycin-labeling doxycycline-inducible λN-3 × HA-turboID construct and a G418-labeled pCGN-boxB-KLF16-FL-boxB-GFP construct. These expression vectors were transiently transfected into HEK293T cells for viral packaging. T24 cells were then infected with the λN-3 × HA-turboID lentivirus, and stable cell lines expressing λN-3 × HA-turboID were established through puromycin selection. A secondary infection was performed using the boxB-KLF16-FL-boxB lentivirus or a boxB-vector control. G418 selection was added to obtain the stable cell line of interest. Subsequently, stable cells were treated with doxycycline (100 ng mL^−1^) for 24 h, followed by the addition of 50 μM biotin to the culture medium for 20 min. The cells were lysed and sonicated in RIPA-SDS buffer (50 mM Tris–HCl, pH 8.0; 150 mM NaCl; 0.1% SDS; 0.5% sodium deoxycholate; 1% Triton X-100). The clarified cell lysates were incubated with streptavidin beads at 4 °C for 2 h, followed by sequential washes: twice with lysis buffer, once each with 1 M KCl buffer, 0.1 M Na_2_CO_3_ buffer, 2 M urea in 10 mM Tris buffer, and twice more with lysis buffer. After suspension in 1 × loading buffer and boiling at 100 °C for 10 min, the beads were subjected to mass spectrometry (MS) analysis.

### RNA interference treatment

BLCA cells, at 20–30% confluence, were transfected with 100 nM small interfering RNAs (siRNAs) using Lipofectamine RNAiMAX in accordance with the manufacturer’s instructions. The sequences of all siRNAs used in this study are listed in Supplementary Material 2.

### Ubiquitination assay

UM-UC-3 stable cells were seeded in 10 cm plates and transfected with a His-tagged ubiquitin (His-Ub) plasmid for 24 h. Cells were treated with bortezomib (1 μM) for 8 h and subsequently lysed with buffer A (0.1 M Na_2_HPO_4_/NaH_2_PO_4_; 6 M guanidine-HCl; 10 mM imidazole, pH 8.0). After sonication, the cell lysates were incubated with Ni–NTA beads (Beyotime) at room temperature for 2–4 h. His-ubiquitinated precipitates were washed three times with buffer A, twice with buffer A&Ti (3 parts buffer Ti and one part buffer A), and once with buffer Ti (20 mM imidazole and 25 mM Tris–HCl, pH 6.8). The His-ubiquitinated precipitates were then resuspended in buffer Ti and boiled at 100 °C for 10 min after adding 1 × SDS sample buffer.

### Immunohistochemical staining (IHC) and fluorescence IHC

Paraffin-embedded tissue slides were heated at 60 °C for 2 h, followed by deparaffinization, dehydration, and antigen retrieval. Subsequently, the tissue slides were blocked with goat serum (30 min, room temperature) and then incubated with the indicated primary antibodies against KLF16 (sc-377519, SantaCruz) and MYC (ab32072, Abcam) at 4 °C overnight. The tissue slides were then washed three times with PBST and treated with 3% H_2_O_2_ for 10 min to block endogenous peroxidase activity. Afterwards, the tissue slides were incubated with anti-mouse/rabbit IgG secondary antibody, treated with DAB reagent (Dako Omnis), and counterstained with hematoxylin staining solution (Beyotime Biotechnology) according to the manufacturer’s instructions. The expression of KLF16 and MYC was evaluated using the H-score, which combines staining intensity (0, no evidence of staining; 1, weak; 2, moderate; 3, strong) with the percentage of stained cells at each intensity level (0% to 100%). The final H-score was calculated by multiplying the staining intensity by the percentage of cells, as previously reported [54]. The median IHC score of KLF16 served as the cutoff value to distinguish between the high and low KLF16 expression groups. The IHC scores of MYC and KLF16 are listed in Supplementary Material 5. The paraffin-embedded pathologic specimens from 80 patients with BLCA were obtained from Sun Yat-sen University Cancer Center, and additional details are provided in Supplementary Material 6.

For fluorescence IHC, slides were subjected to deparaffinization, antigen retrieval, and blocking. They were then incubated with primary antibodies against KLF16 and MYC overnight at 4 °C. Afterward, the slides were treated with Alexa Fluor 594/488-conjugated secondary antibody (Invitrogen) for 2 h at room temperature. Cell nuclei were counterstained with Hoechst 33,342 (Thermo Fisher, H3570) for 2 min. The antibodies used and their concentrations are detailed in Supplementary Material 3.

### RNA-seq analysis

T24 cells stably expressing KLF16-targeted sgRNAs/shRNAs or a control were collected, and total RNA was extracted using TRIzol reagent (Life Technologies, 15,596,026). RNA sequencing assays were performed by Novogene (Beijing, China). Genes with an adjusted *P*-value < 0.05 were considered differentially expressed genes. For GSEA, the MYC signaling pathway gene set was generated based on the gene list from the KEGG. Normalized expression data were then analyzed and visualized using GSEA software (version 3.0, http://www.broadinstitute. org/gsea/). The RNA-seq analysis results for T24 cells expressing KLF16-targeted shRNAs are detailed in Supplementary Material 7.

### Chromatin immunoprecipitation (ChIP)-qPCR

Cells were cross-linked with 1% formaldehyde (10 min, room temperature) and stopped with glycine (125 mM) for 5 min at room temperature. After four times washes with cold PBS, cells were scraped from the dishes and centrifuged at 800 × g at 4 °C for 5 min. Fixed pellet cells were lysed with cell lysis buffer (20 mM Tris–HCl, pH 8.0; 85 mM KCl; 0.5% NP-40; protease inhibitor cocktails) on ice for 15 min. Supernatants were removed, and the isolated nuclei were resuspended in nuclei lysis buffer (10 mM Tris–HCl, pH 7.5; 1% NP40; 0.5% deoxycholate and 0.1% SDS; protease inhibitor cocktails). After sonication, the samples were immunoprecipitated with MYC antibody (2 μg, ab32072, Abcam) or normal rabbit IgG (CST) overnight at 4 °C. ChIP-grade Protein A/G magnetic beads (Millipore) were added and incubated for 2 h. The immunoprecipitates were then washed twice, each with low-salt, high-salt, LiCl buffer, and TE buffer. Eluted DNA was reverse-crosslinked and purified using a PCR purification kit (Qiagen), and analyzed by qPCR on the LightCycler 480 Instrument II system (Roche). The specific primers used are provided in Supplementary Material 2.

### CUT&Tag assays

CUT&Tag assays were performed essentially as described previously [55]. Briefly, 1 × 10^5^ T24 cells were incubated with 10 μL of concanavalin A-coated magnetic beads (Bangs Laboratories) for 10 min at room temperature. Bead-bound cells were then suspended in dig wash buffer (20 mM HEPES, pH 7.5; 150 mM NaCl; 0.5 mM Spermidine; 1 × Protease inhibitor cocktail; 0.05% Digitonin; and 2 mM EDTA) and incubated overnight at 4 °C with a 1:50 dilution of antibodies against MYC (ab32072, Abcam) and FLAG (14,793, CST). After removal of primary antibody using a magnet stand, cells were incubated with a secondary antibody (1:100 Anti-Rabbit IgG antibody, Goat monoclonal: Millipore AP132) for one hour at room temperature, followed by incubation with the pA-Tn5 adapter complex for an additional hour. After three washes with Dig-med buffer (0.01% digitonin; 20 mM Hepes, pH 7.5; 300 mM NaCl; 0.5 mM spermidine; and 1 × protease inhibitor cocktail), cells were resuspended in Tagmentation buffer (10 mM MgCl_2_ in Dig-med Buffer) and incubated at 37 °C for 1 h. Genomic DNA was extracted using the TIANamp Genomic DNA Kit (TIANGEN) according to the manufacturer’s instructions. Subsequently, sequencing libraries were purified using XP beads (Beckman Counter). The sequencing procedure was carried out on the Illumina NovaSeq 6000 platform, utilizing PE150 sequencing as recommended by the manufacturer. The antibodies used and their concentrations are listed in Supplementary Material 3.

### Animal experiments

Male athymic BALB/c nude mice were purchased from the GemPharmatech. For the BLCA subcutaneous transplantation model, T24 (3 × 10^6^) and BIU87 (4 × 10^6^) stable cells were resuspended in PBS and mixed in equal volumes with matrigel. The mixture was then injected subcutaneously into the flanks of the nude mice at the age of 5 weeks (*n* = 7–8 per group). Seven days after injection, tumor sizes were measured using a caliper every three days, and tumor volume was calculated using the formula: tumor volume = 1/2 (length × width^2^). Mice were sacrificed at the final time point, and the xenograft tumors were dissected and weighed.

For assays involving DDP/gemcitabine and OTX015 combination treatments, mice bearing T24 tumors were randomly assigned to the experimental groups. The groups received either vehicle, DDP (5 mg kg^−1^, intraperitoneally twice weekly), gemcitabine (50 mg kg^−1^, intraperitoneally twice weekly), OTX015 (25/50 mg kg^−1^, intragastric administration daily), or a combination of DDP/gemcitabine plus OTX015.

For combination treatment assays with DDP and ABBV-744, mice bearing T24 tumors were randomly assigned to 6 groups (*n* = 7 per group). The groups received either vehicle, DDP (3/5 mg kg^−1^, intraperitoneally twice weekly), ABBV-744 (10 mg kg^−1^, intragastric administration daily), or a combination of DDP and ABBV-744. The vehicle was prepared and administered in the same manner as the drug treatments, with the exception that no drug was added. Doses of 100 μL were administered by intraperitoneal injection or intragastric administration. Tumor sizes were measured and tumor volume was calculated. Mice were sacrificed at the final time point, and the xenograft tumors were dissected and weighed.

### Statistical analysis

All results were obtained from three independent experiments. Statistical analyses were performed with GraphPad Prism 10.2.0 using Student’s two-tailed unpaired *t*-tests for comparisons between two groups. KLF16 and MYC expression and cancer survival curves were assessed by Kaplan–Meier plots and compared by a log-rank test. To test differences in mouse tumor growth, we conducted a two-way ANOVA analysis followed by Tukey’s multiple comparisons test. To determine if age, gender, grade, TNM stage, and treatment are independent risk parameters from clinical features, we performed multivariate analyses using the Cox proportional hazards model and data stratification. Statistical research was carried out using the SPSS 26.0 software (SPSS, Inc., Chicago, IL, USA). Data are presented as the mean ± SD from a minimum of three independent experiments. For correlation analysis, the Spearman correlation coefficient was calculated for crosstabs and the *P*-value was determined by Pearson's chi-squared test.

## Supplementary Information

Supplementary Material 1.

Supplementary Material 2.

Supplementary Material 3.

Supplementary Material 4.

Supplementary Material 5.

Supplementary Material 6.

Supplementary Material 7.

## Data Availability

RNA-sequencing data from this study have been deposited in the Genome Sequence Archive for Humans with the accession code HRA009231. Mass spectrometry data have been submitted to the ProteomeXchange Consortium via the iProX partner repository [56] with the accession code PXD057733. ChIP-seq data of KLF16 and MYC (ENCSR009KLQ, ENCSR000DMM, ENCSR784BVD, ENCSR000FAG, ENCSR000EZD) were retrieved from the ENCODE database (https://www.encodeproject.org/). All other data generated in this study are available within the article and its supplementary materials.

## Abbreviations

BLCA: Bladder cancer
PFS: Progression-free survival
CSS: Cancer-specific survival
CI: Confidence interval
DUSP16: Dual-specificity phosphatase 16
WBP11: WW domain binding protein 11
DDP: Cisplatin
TAD: Transactivation domain
CDKs: Cyclin-dependent kinases
BRD4: Bromodomain-containing 4
KLF16: Krüppel-like factor 16
ERK: Extracellular signal-regulated kinases
BET: Bromodomain and extraterminal
sgRNA: Single guide RNA
IHC: Immunohistochemistry
TMA: Tissue microarray
KO: Knock out
KD: Knockdown
GSEA: Gene set enrichment analyses
DEGs: Differentially expressed genes
TCGA: The Cancer Genome Atlas
CMG: CDC45-MCM2-7-GINS
MCM2-7: Minichromosome maintenance protein 2–7
CDC45: Cell division cycle protein 45
qPCR: Quantitative polymerase chain reaction
CUT&Tag: Cleavage Under Targets and Tagmentation
WT: Wild-type
LncRNAs: Long non-coding RNAs
RIP: RNA immunoprecipitation
CTD: C-terminal domain
ENCODE: Encyclopedia of DNA Elements
IDRs: Intrinsically disordered regions
mEGFP: Monomeric enhanced green fluorescent protein
Pol II-CTD: Polymerase II C-terminal domain
SRA: Steroid receptor RNA activator
ATCC: American Type Culture Collection
PDC: Patient-derived cell line
STR: Short tandem repeat
PEI: Polyethyleneimine
PBS: Phosphate-buffered saline
OD: Optical density
MS: Mass spectrometry
ChIP: Chromatin immunoprecipitation
IC50: Halfmaximal inhibitory concentration
