# Correction: The KLF16/MYC feedback loop is a therapeutic target in bladder cancer

**DOI:** 10.1186/s13046-026-03744-0

**Published:** 2026-05-27

**Authors:** Lisi Zheng, Jingxuan Wang, Shan Han, Li Zhong, Zefu Liu, Bin Li, Ruhua Zhang, Liwen Zhou, Xianchong Zheng, Zhenhua Liu, Cuiling Zeng, Ruonan Li, Yezi Zou, Liqin Wang, Yuanzhong Wu, Tiebang Kang

**Affiliations:** 1 Sun Yat-Sen University Cancer Center, State Key Laboratory of Oncology in South China, Guangdong Provincial Clinical Research Center for Cancer, 651 Dongfeng Road East, Guangzhou, 510060 People’s Republic of China; 2 Center of Digestive Disease, Scientific Research Center, The Seventh Affiliated Hospital, Sun Yat-Sen University, Shenzhen, 518107 People’s Republic of China; 3 Department of Urology, Xiangya Hospital, Central South University, Changsha, 410008 People’s Republic of China; 4 MOE Key Laboratory of Gene Function and Regulation, State Key Laboratory of Biocontrol, School of Life Sciences, Sun Yat-Sen University, Guangzhou, 510275 People’s Republic of China

**Correction: J Exp Clin Cancer Res 43, 303 (2024)**

**https://doi.org/10.1186/s13046-024-03224-3**

Following publication of the original article [1], the authors found errors in the published version of Figure 7 and Figure 8. In Figure 7k, the immunohistochemical image of MYC for Case 1 was inadvertently used while in Figure 8I, a p-value was inadvertently omitted during the figure preparation process.

**Incorrect Figure 7**

**Fig. 7:**
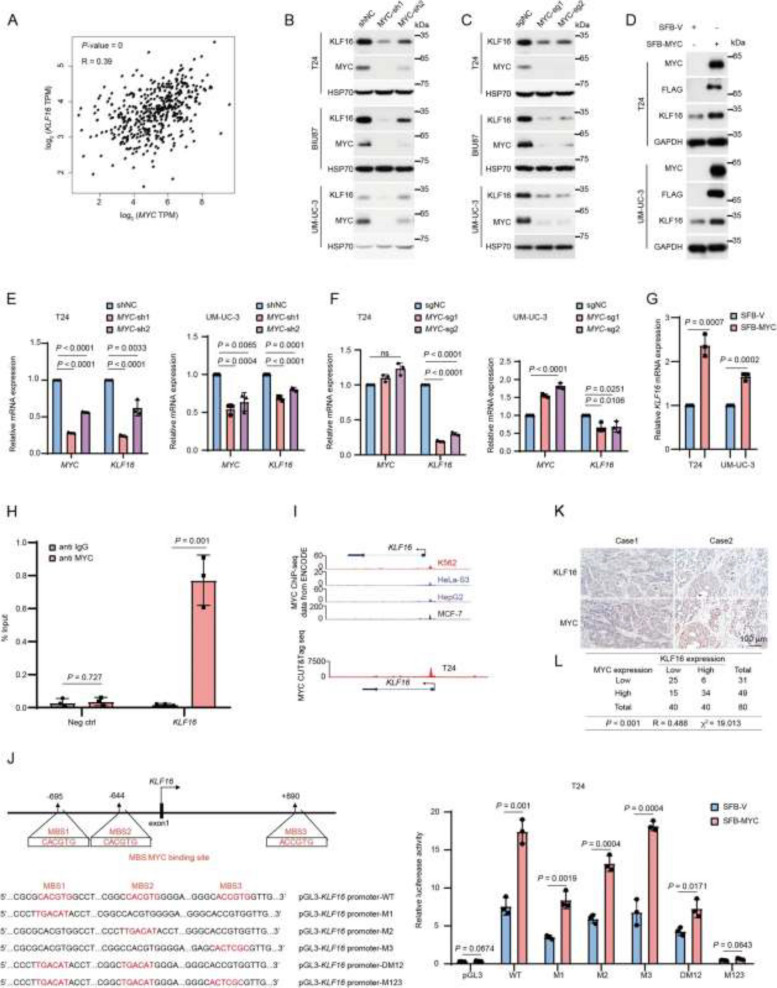
MYC transcriptionally upregulates *KLF16*. **A** The scatter plot shows the Pearson correlation of *MYC* and *KLF16* mRNA expression in BLCA tissues. Data from GEPIA database: http://gepia.cancer-pku.cn/index.html. *P* value was determined by the log-rank test and the *R*-value was analyzed using Spearman’s correlation test. **B**-**G** The BLCA cell lines with MYC depletion or overexpression were analyzed by Western blotting (**B**-**D**) and qPCR (**E**–**G**). *n* = 3 biologically independent experiments. **H** ChIP-qPCR analysis of MYC occupancy at *KLF16* promoter region in T24 cells. Neg ctrl, negative control. *n* = 3 biologically independent experiments. **I** Track view of MYC ChIP-seq density profile on KLF16 genomic region in the indicated cell lines from published data sets displayed by UCSC Genome Browser: http://genome.ucsc.edu [36] (upper panel). MYC CUT&Tag seq tracks in gene loci of *KLF16* in T24 cells displayed by IGV software (lower panel). ChIP-seq data from the ENCODE database (https://www.encodeproject.org). **J** Schematic presentation of MYC binding sites on the KLF16 locus (left panel). T24 cells expressing SFB-MYC were transfected with the indicated wild-type (WT) or mutants of KLF16 promoter, along with the Renilla control reporter for 24 h. Then, cells were analyzed for the relative luciferase activity (right panel). MBS, MYC binding site. **K** Representative immunohistochemical images of both KLF16 and MYC from 80 paraffin-embedded BLCA tissues. Scale bar, 100 μm. **L** Crosstab shows the distribution of cancer tissues in the bladder cancer tissues used in (**K**) according to the median H-Score of KLF16 and MYC. The *P* value and chi-square were analyzed using Pearson’s chi-squared test, and the *R*-value was analyzed using Spearman’s correlation test. All error bars represent mean ± SD and *P* values were calculated using two-tailed unpaired Student’s *t*-tests unless noted otherwise

**Correct Figure 7**

**Fig. 7:**
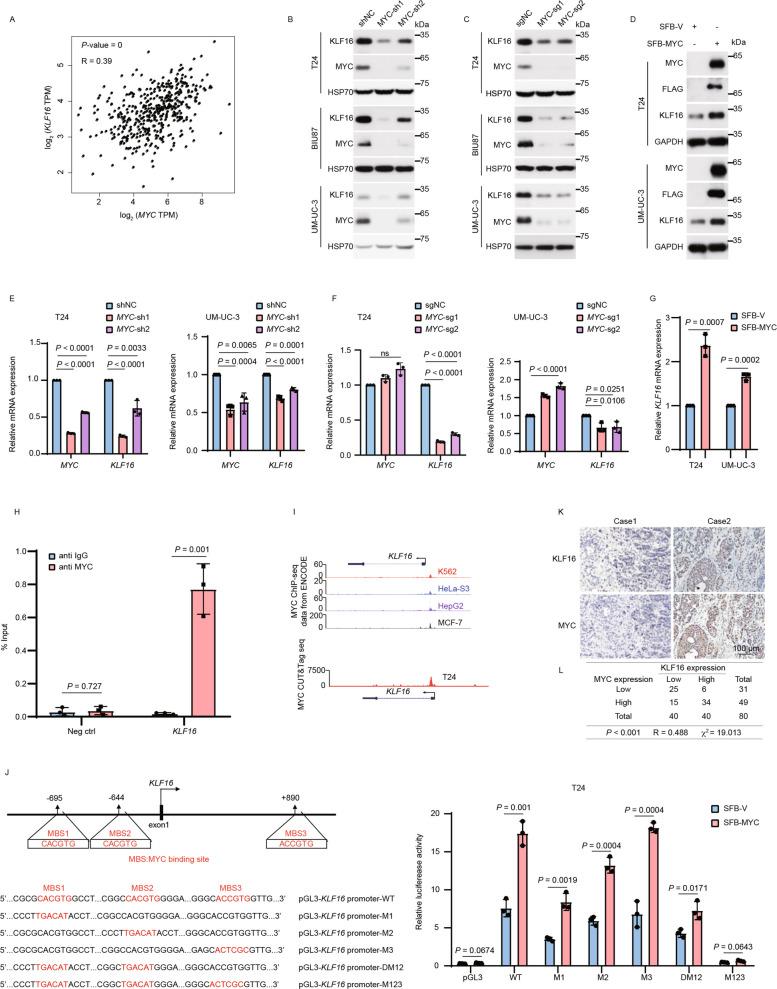
MYC transcriptionally upregulates *KLF16*. **A** The scatter plot shows the Pearson correlation of *MYC* and *KLF16* mRNA expression in BLCA tissues. Data from GEPIA database: http://gepia.cancer-pku.cn/index.html. *P* value was determined by the log-rank test and the *R*-value was analyzed using Spearman’s correlation test. **B**-**G** The BLCA cell lines with MYC depletion or overexpression were analyzed by Western blotting (**B**-**D**) and qPCR (**E**–**G**). *n* = 3 biologically independent experiments. **H** ChIP-qPCR analysis of MYC occupancy at *KLF16* promoter region in T24 cells. Neg ctrl, negative control. *n* = 3 biologically independent experiments. **I** Track view of MYC ChIP-seq density profile on KLF16 genomic region in the indicated cell lines from published data sets displayed by UCSC Genome Browser: http://genome.ucsc.edu [36] (upper panel). MYC CUT&Tag seq tracks in gene loci of *KLF16* in T24 cells displayed by IGV software (lower panel). ChIP-seq data from the ENCODE database (https://www.encodeproject.org). **J** Schematic presentation of MYC binding sites on the KLF16 locus (left panel). T24 cells expressing SFB-MYC were transfected with the indicated wild-type (WT) or mutants of KLF16 promoter, along with the Renilla control reporter for 24 h. Then, cells were analyzed for the relative luciferase activity (right panel). MBS, MYC binding site. **K** Representative immunohistochemical images of both KLF16 and MYC from 80 paraffin-embedded BLCA tissues. Scale bar, 100 μm. **L** Crosstab shows the distribution of cancer tissues in the bladder cancer tissues used in (**K**) according to the median H-Score of KLF16 and MYC. The *P* value and chi-square were analyzed using Pearson’s chi-squared test, and the *R*-value was analyzed using Spearman’s correlation test. All error bars represent mean ± SD and *P* values were calculated using two-tailed unpaired Student’s *t*-tests unless noted otherwise

**Incorrect Figure 8**

**Fig. 8:**
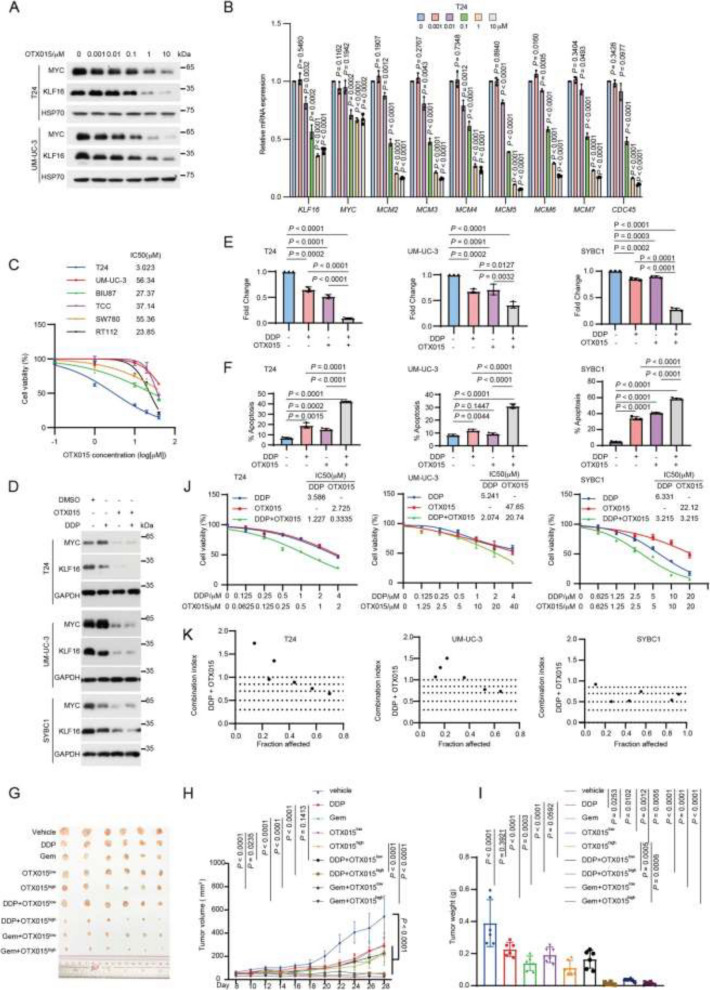
OTX015 suppresses tumor growth and enhances chemotherapy sensitivity in BLCA. **A** T24 and UM-UC-3 cells were treated with increasing concentrations of OTX015 for 48 h and then were analyzed by Western blotting. **B** T24 cells in (**A**) were subjected to qPCR analysis for the indicated mRNA levels. **C** The indicated bladder cancer cell lines were treated with the indicated concentration of OTX015 (0, 0.1 μM, 1 μM, 10 μM, 20 μM, 40 μM) for 48 h, and then the relative cell growth rates were determined by MTT assay. *n* = 3 biologically independent experiments. **D**-**F** T24, UM-UC-3 and SYBC1 cells were treated with DMSO, OTX015 (10 μM for UM-UC-3 and SYBC1, 1 μM for T24) or DDP (2.5 μM) separately or together, the indicated proteins were determined by Western blotting (**D**), colony formation (**E**) and flow cytometry analysis of apoptosis (**F**). *n* = 3 biologically independent experiments. **G**-**I** Mice bearing T24 tumors were randomly divided into the indicated groups (*n* = 6 mice per group). DDP (5 mg kg−1) or gemcitabine (Gem, 50 mg kg−1) were injected intraperitoneally twice weekly. OTX015 (25 mg kg−1 & 50 mg kg−1) were daily given via intragastric administration for about 3 weeks. Tumor volumes (**H**) and tumor weights (**I**) were measured. **J** T24, UM-UC-3 and SYBC1 cells were treated with the indicated concentration of OTX015 or DDP alone or in combination for 48 h, and then the relative cell growth rates were determined by MTT assays. *n* = 3 biologically independent experiments. **K** The Combination Index (CI) of OTX015 and DDP was calculated based on results from (**J**) by using CalcuSyn. CI < 1, = 1, and > 1 indicate synergism, additive effect, and antagonism, respectively. All error bars represent mean ± SD. *P* values in **H** were calculated by two-way ANOVA with Tukey’s multiple comparisons test. *P* values in **B**, **E**–**F**, **I** were calculated using two-tailed unpaired Student’s *t*-tests

**Correct Figure 8**

**Fig. 8:**
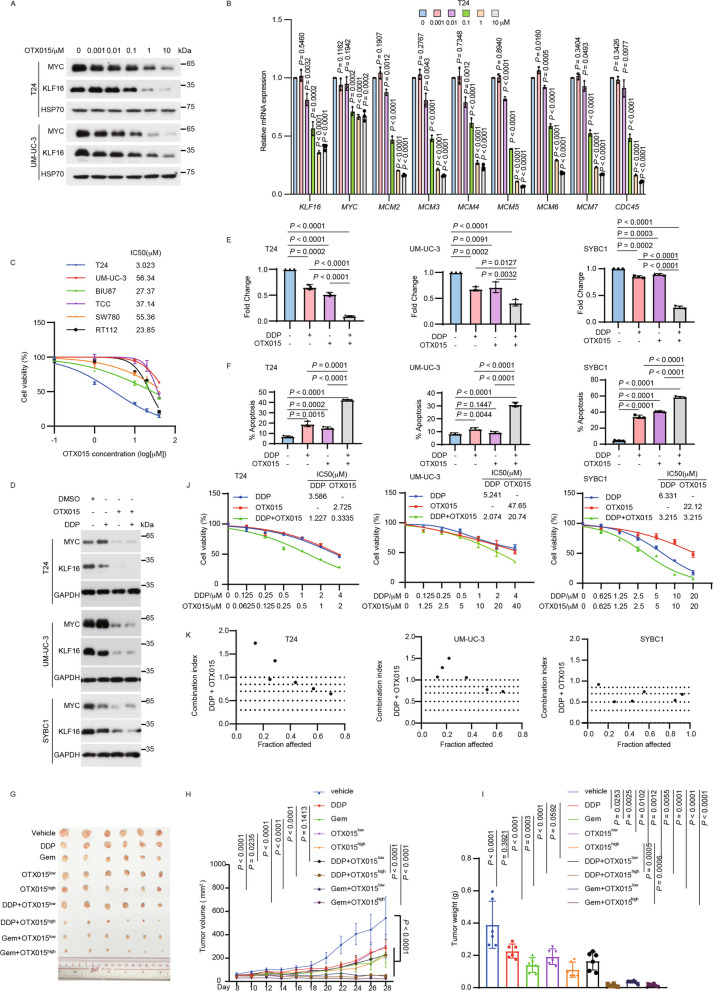
OTX015 suppresses tumor growth and enhances chemotherapy sensitivity in BLCA. **A** T24 and UM-UC-3 cells were treated with increasing concentrations of OTX015 for 48 h and then were analyzed by Western blotting. **B** T24 cells in (**A**) were subjected to qPCR analysis for the indicated mRNA levels. **C** The indicated bladder cancer cell lines were treated with the indicated concentration of OTX015 (0, 0.1 μM, 1 μM, 10 μM, 20 μM, 40 μM) for 48 h, and then the relative cell growth rates were determined by MTT assay. *n* = 3 biologically independent experiments. **D**-**F** T24, UM-UC-3 and SYBC1 cells were treated with DMSO, OTX015 (10 μM for UM-UC-3 and SYBC1, 1 μM for T24) or DDP (2.5 μM) separately or together, the indicated proteins were determined by Western blotting (**D**), colony formation (**E**) and flow cytometry analysis of apoptosis (**F**). *n* = 3 biologically independent experiments. **G**-**I** Mice bearing T24 tumors were randomly divided into the indicated groups (*n* = 6 mice per group). DDP (5 mg kg−1) or gemcitabine (Gem, 50 mg kg−1) were injected intraperitoneally twice weekly. OTX015 (25 mg kg−1 & 50 mg kg−1) were daily given via intragastric administration for about 3 weeks. Tumor volumes (**H**) and tumor weights (**I**) were measured. **J** T24, UM-UC-3 and SYBC1 cells were treated with the indicated concentration of OTX015 or DDP alone or in combination for 48 h, and then the relative cell growth rates were determined by MTT assays. *n* = 3 biologically independent experiments. **K** The Combination Index (CI) of OTX015 and DDP was calculated based on results from (**J**) by using CalcuSyn. CI < 1, = 1, and > 1 indicate synergism, additive effect, and antagonism, respectively. All error bars represent mean ± SD. *P* values in **H** were calculated by two-way ANOVA with Tukey’s multiple comparisons test. *P* values in **B**, **E**–**F**, **I** were calculated using two-tailed unpaired Student’s *t*-tests

The original article [1] has been corrected.
