# Supplementary material for: The KLF16/MYC feedback loop is a therapeutic target in bladder cancer
Source: J Exp Clin Cancer Res. 2024 Nov 18;43:303. doi: 10.1186/s13046-024-03224-3 (PMC11571712; doi:10.1186/s13046-024-03224-3)
Supplement: Supplementary file 1 — Supplementary Material 1. [file 13046_2024_3224_MOESM1_ESM.docx]

**Supplementary Material 1**

**The KLF16/MYC feedback loop is a therapeutic target in bladder cancer**

# Lisi Zheng, Jingxuan Wang, Shan Han, Li Zhong, Zefu Liu, Bin Li, Ruhua Zhang, Liwen Zhou, Xianchong Zheng, Zhenhua Liu, Cuiling Zeng, Ruonan Li, Yezi Zou, Liqin Wang*, Yuanzhong Wu*, and Tiebang Kang*

**Address Correspondence to:** *Tiebang Kang, Tel: 86-20-8734-3183, Fax: 86-20-8734-3170; E-mail: [kangtb@sysucc.org.cn](mailto:kangtb@sysucc.org.cn). or *Yuanzhong Wu, Tel: 86-20-8734-3183, Fax: 86-20-8734-3170; E-mail: [wuyzh@sysucc.org.cn](mailto:wuyzh@sysucc.org.cn) State Key Laboratory of Oncology in South China, Sun Yat-sen University Cancer Center, 651 Dongfeng Road East, Guangzhou, China, 510060 or *Liqin Wang, Tel: 86-20-87340285, Fax: 86-20-8734-3170; E-mail: [wanglq1@sysucc.org.cn](mailto:wanglq1@sysucc.org.cn) State Key Laboratory of Oncology in South China, Sun Yat-sen University Cancer Center, OneHub GKC, F-804, Sino-Singapore Knowledge City, HuangPu District, Guangzhou, China, 510555.

**Supplemental Figures.**


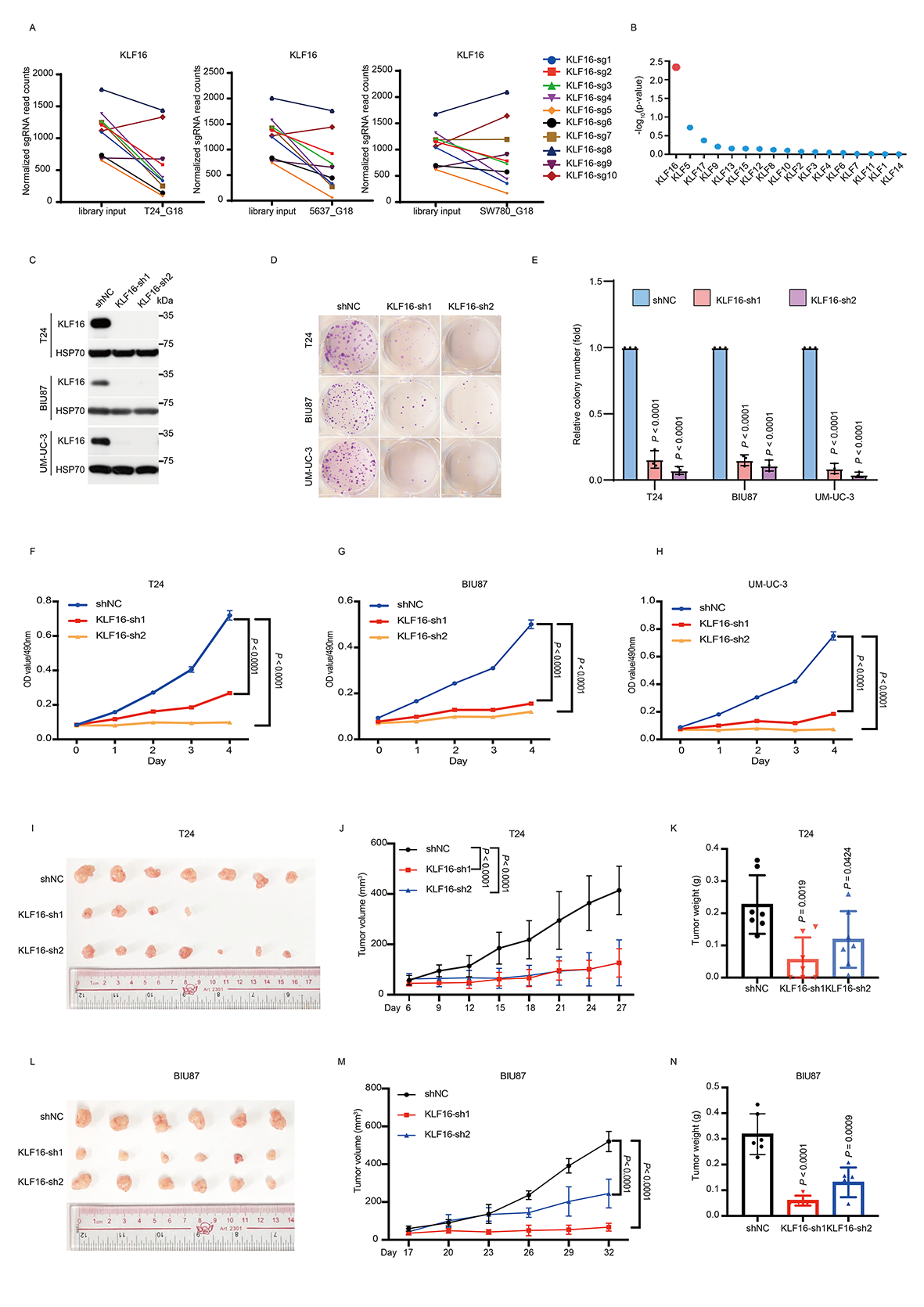


**Figure. S1. KLF16 promotes tumor growth in BLCA.**

**(A**). Read counts of individual sgRNAs targeting KLF16 were shown**. (B)**. CRISPR screening results analyzed by MAGeCK of KLF family member genes. **(C).** Western blotting of the indicated proteins in T24, BIU87 and UM-UC-3 cells expressing *KLF16-*targeted shRNAs. **(D-E).** Representative images **(D)** and quantification **(E)** of colonies formed by the indicated BLCA cells in **(C)**. The colony numbers were quantified using ImageJ software. *n* = 3 biologically independent experiments. **(F-H).** Cell viability of the indicated stable cells in **(C)** was measured by MTT assay at the indicated time points. *n* = 3 biologically independent experiments. **(I-N).** T24 and BIU87 cells expressing KLF16-targeted shRNAs were subcutaneously injected into nude mice. Representative images of subcutaneous xenograft tumors were shown **(I and L)**. Tumor volumes were measured at the indicated time points **(J and M)**. Tumor weights were measured at the end point **(K and N)**. *n* = 7 nude mice per group were used for T24 experiments, and *n* = 6 nude mice per group were used for BIU87 experiments. All error bars represent mean ± SD and *P* values in **E-H**, **K** and **N** were calculated using two-tailed unpaired Student’s *t*-tests. *P* values in **J** and **M** were calculated by two-way ANOVA analysis followed by Tukey’s multiple comparisons test.


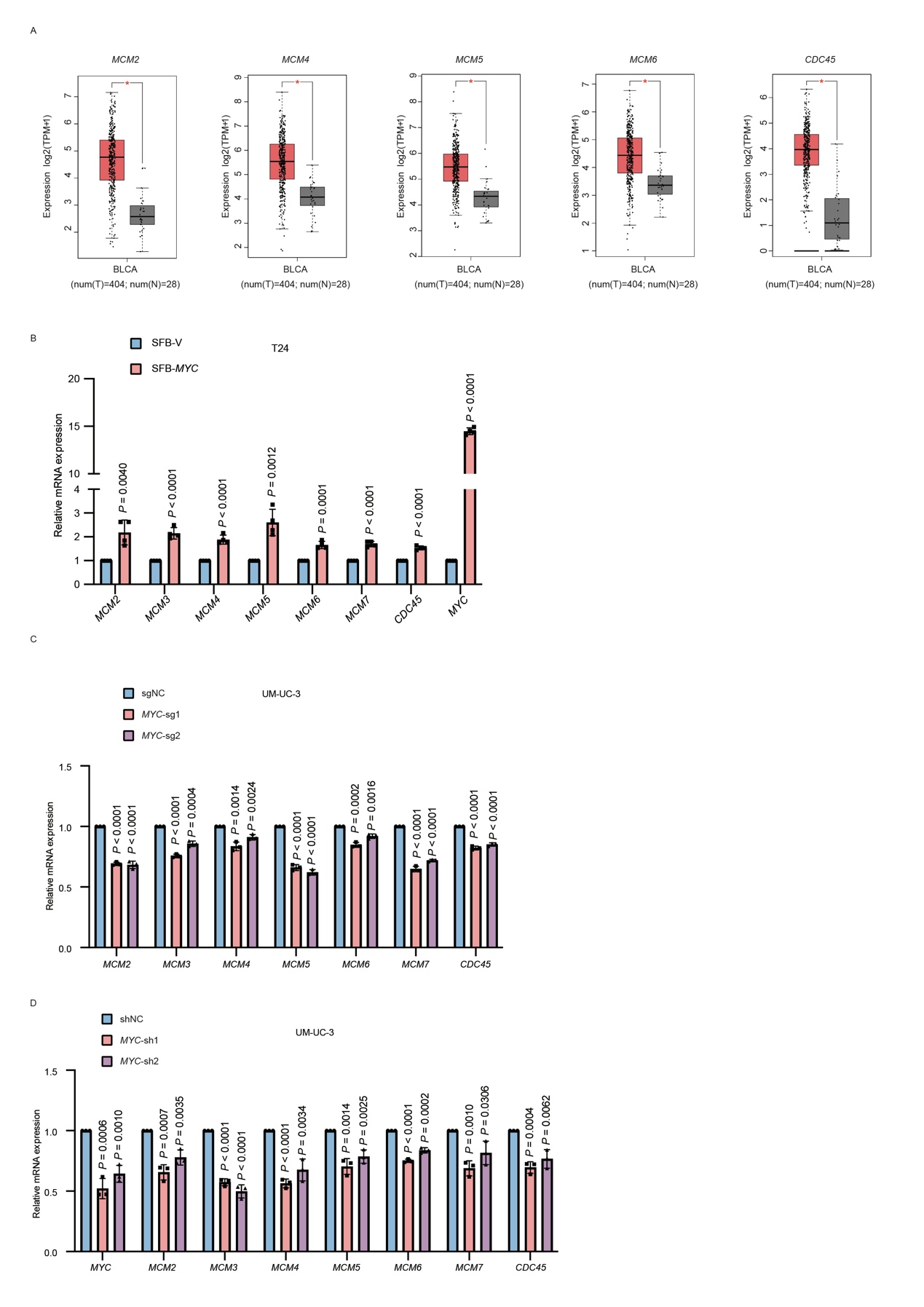


**Figure. S2. MYC promotes MCM2-7 and CDC45 transcription in BLCA.**

**(A**). Box plots demonstrate the relative mRNA expression levels (log2 (TPM+1)) of *MCM2*, *MCM4*, *MCM5*, *MCM6*, and *CDC45* in BLCA tumor (T) (red column) and normal (N) (grey column) tissues. Data from Gene Expression Profiling Interactive Analysis (GEPIA) database: <http://gepia.cancer-pku.cn/index.html>. Single asterisk represents significant difference (*P* < 0.05). **(B-D).** qRT–PCR analyzing the mRNA levels of *MYC* and MYC target genes in T24 cells stably expressing SFB-MYC **(B)** and UM-UC-3 cells stably expressing negative control (NC) or sgMYC **(C)** or shMYC **(D)**.

All error bars represent mean ± SD and *P* values were calculated using two-tailed unpaired Student’s *t*-tests.


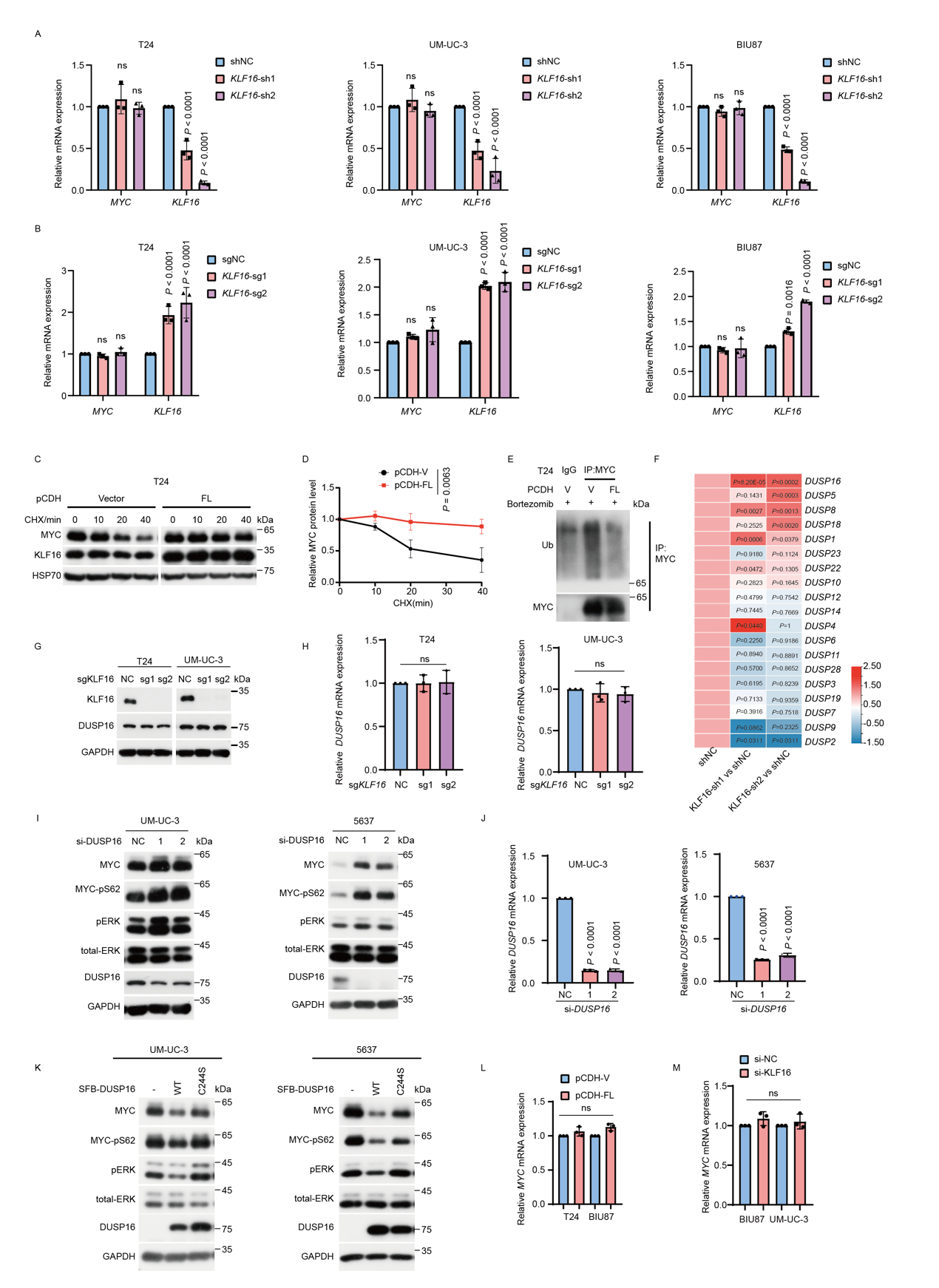


**Figure. S3.** ***KLF16* mRNA stabilizes MYC protein via destabilizing *DUSP16* mRNA in BLCA.**

**(A-B).** qRT–PCR analyzing the mRNA levels of *MYC* and *KLF16* in the indicated cells stably expressing KLF16-targeted shRNAs **(A)** or sgRNAs **(B)**. *n* = 3 biologically independent experiments. **(C-D)**. T24 cells stably expressing vector or KLF16 full length (FL) mRNA were treated with 20 μg mL^-1^ cycloheximide (CHX) at the indicated time points and then analyzed by Western blotting **(C)**. Quantitation of MYC protein levels was based on the Western blotting results **(D)**. *n* = 3 biologically independent experiments. **(E)** T24 cells stably expressing vector or KLF16 FL mRNA were incubated with bortezomib (1 μM) for 8 h, and then subjected to IP with anti-MYC antibody followed by Western blotting to detect MYC’s ubiquitination level. **(F)**. The heatmap shows relative expression levels of DUSP family members in the indicated T24 stable cells. The *P* values were adjusted using the Benjamini and Hochberg method.

**(G-H)**. T24 and UM-UC-3 cells stably expressing KLF16-targeted sgRNAs were analyzed by Western blotting **(G)** and qRT–PCR **(H)**. *n* = 3 biologically independent experiments. **(I-J)**. UM-UC-3 and 5637 cells transfected with DUSP16-targeted siRNAs for 48h were analyzed by Western blotting **(I)** and qRT–PCR **(J)**. *n* = 3 biologically independent experiments. **(K)**. UM-UC-3 and 5637 cells were transfected with wild-type (WT) or catalytic dead mutant (C244S) SFB-DUSP16 for 48 h followed by Western blotting for the levels of the indicated proteins. **(L).** T24 and BIU87 cells stably overexpression of KLF16 full-length mRNA were analyzed by qPCR. *n* = 3 biologically independent experiments. **(M)** BIU87 and UM-UC-3 cells transfected with KLF16-targeted siRNAs for 48 h were analyzed by qPCR. *n* = 3 biologically independent experiments. All error bars represent mean ± SD and *P* values were calculated using two-tailed unpaired Student’s *t*-tests unless noted otherwise.

**
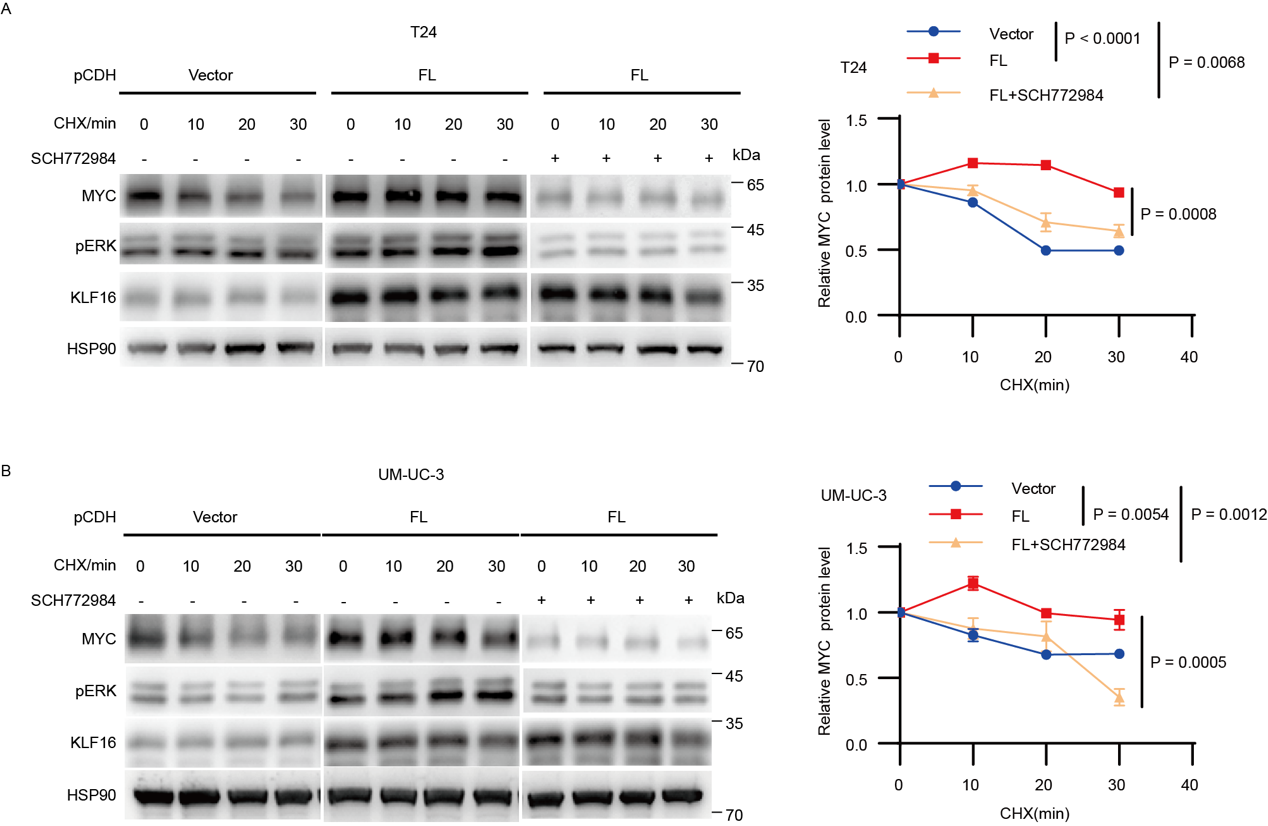
**

**Figure. S4.** **Inhibition of ERK1/2 reduces MYC protein stability in KLF16-overexpressing cells.**

**(A-B).** T24 **(A)** and UM-UC-3 **(B)** cells stably expressing vector or KLF16 full length (FL) mRNA were treated with DMSO or ERK1/2 inhibitor SCH772984 (500 nM) for 60 hours, and then exposed to 20 μg mL^-1^ cycloheximide (CHX) at the indicated time points. Total protein lysates were subjected to Western blotting (left). Quantitation of MYC protein levels was based on the Western blotting results (right). *n* = 3 biologically independent experiments.


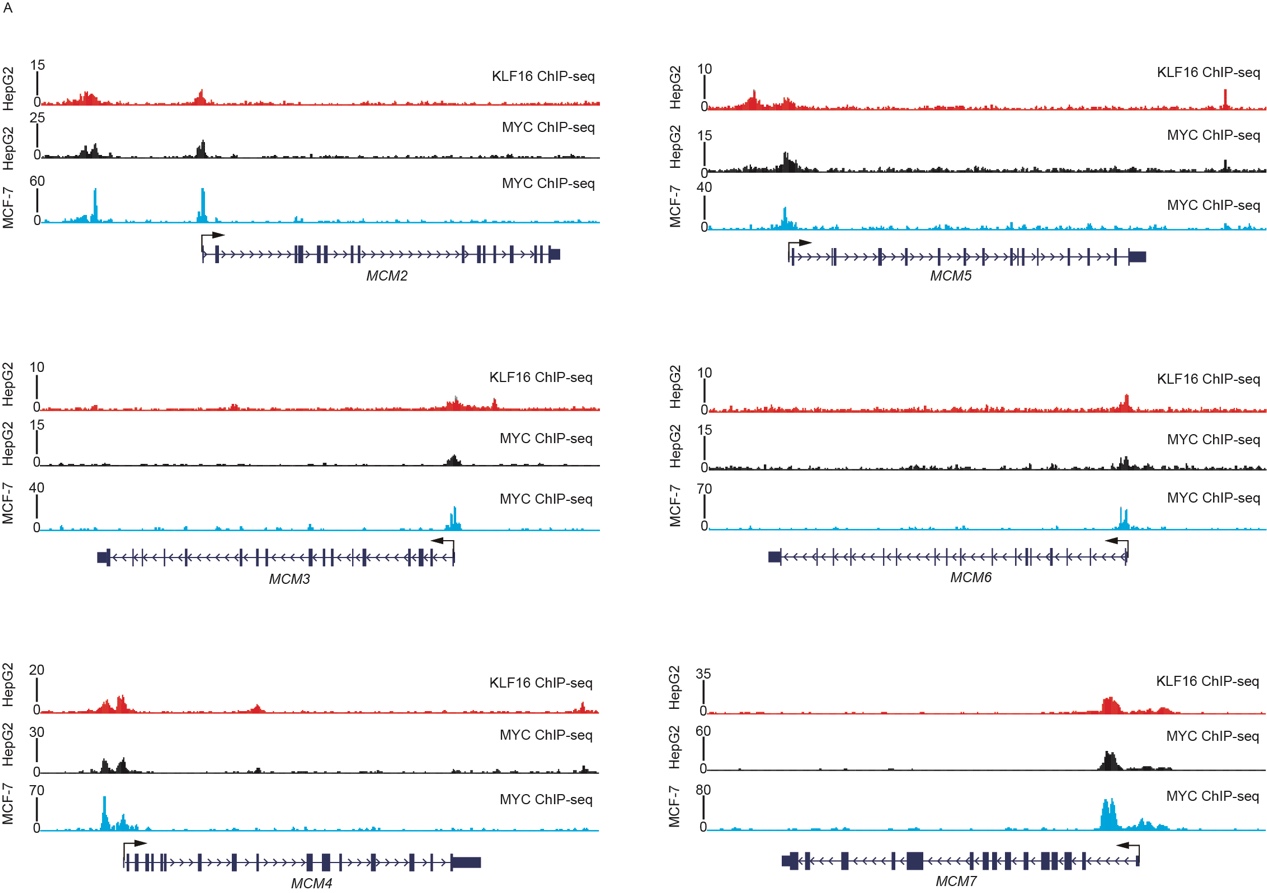


**Figure. S5. Both MYC and KLF16 bound to MCM2-7 genes.**

**(A)**. Track view of KLF16 and MYC ChIP-seq density profile on *MCM2-7* genomic regions in the indicated cell lines from published data sets displayed by UCSC Genome Browser: [http://genome.ucsc.edu](http://genome.ucsc.edu/). Data from the ENCODE database：[https://www.encodeproject.org.](https://www.encodeproject.org/)


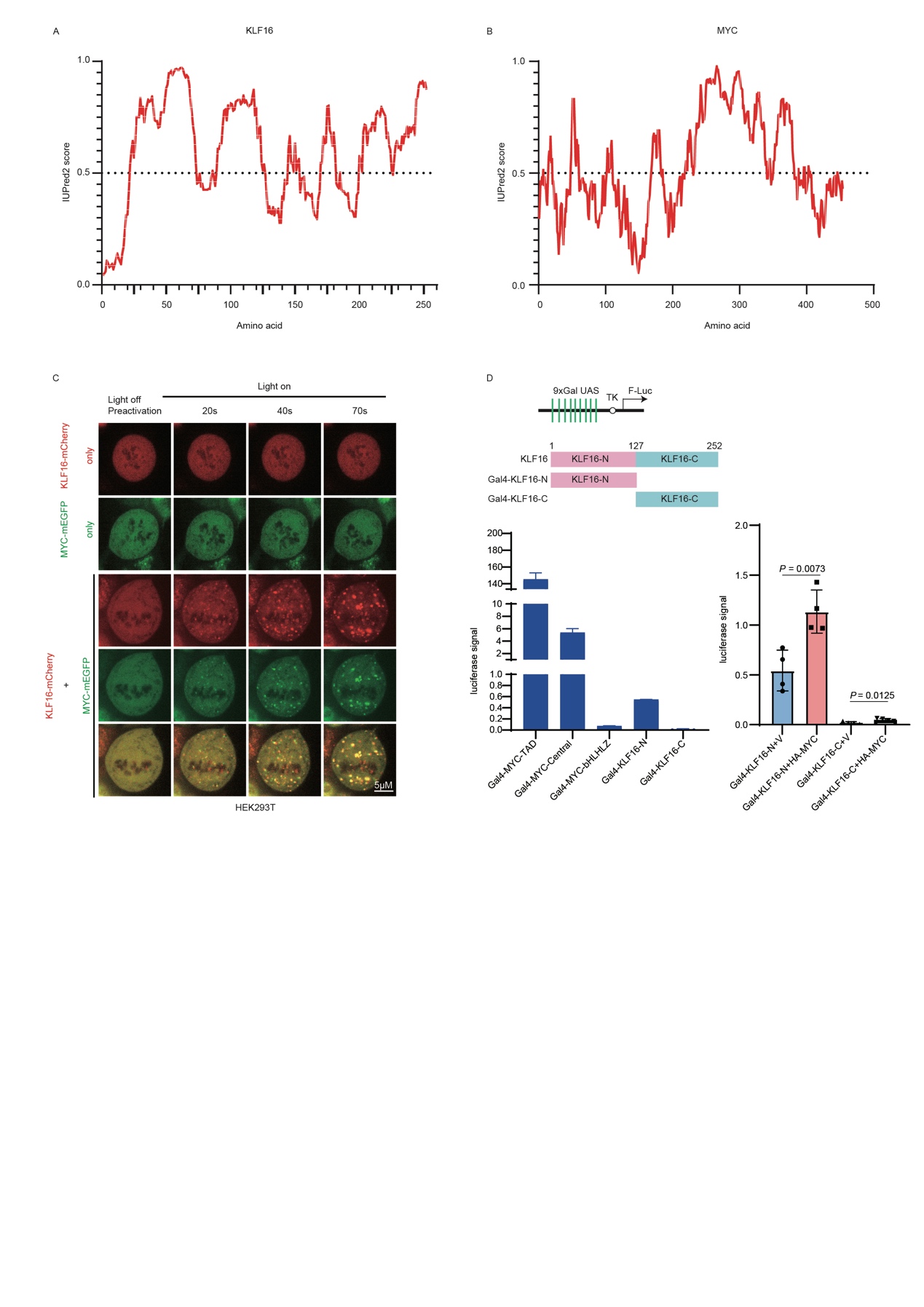


**Figure. S6. KLF16 forms nuclear condensates with MYC to enhance the transcriptional activity of MYC in BLCA.**

**(A-B).** IDR analysis of KLF16 **(A)** and MYC **(B)** using the IUPred2A tool with default parameters. Scores above 0.5 indicate disorder. **(C)**. HEK293T cells were transfected with KLF16-mCherry-Cry2 and MYC-mEGFP-Cry2 separately or together. Images were collected after illumination by a 488 nm laser at the indicated times. **(D)**. Schematic illustration of the 9 x Gal-TK-luciferase reporter system (upper panel) and Gal4-KLF16 truncations (lower panel). HEK293T cells were transfected with the indicated constructs, along with the 9 x Gal-TK-luciferase reporter and the Renilla control reporter for 24 hours. Then, cells were analyzed for the relative luciferase activity. Luc, luciferase

**Figure. S7. OTX015 enhances DDP sensitivity in BLCA.**

**(A)**. UM-UC-3 cells were treated with increasing concentration of OTX015 for 48 hours and then subjected to qRT–PCR analysis for the indicated mRNA levels. **(B)**. T24 and UM-UC-3 cells expressing *KLF16-*targeted shRNAs were treated with the indicated concentration of DDP (0, 0.125 μM, 0.25 μM, 0.5 μM, 1 μM, 2 μM, 4 μM) for 72 hours, and then the cell viability was determined by MTT assays. *n* = 3 biologically independent experiments. **(C)**. Representative images of colonies formed by the indicated BLCA cell lines treated with DMSO, OTX015 (10 μM for UM-UC-3 and SYBC1, 1 μM for T24) or DDP (2.5 μM) separately or together for about 1 week. *n* = 3 biologically independent experiments. **(D)**. Flow cytometry analysis of apoptosis in the indicated cells treated with DMSO, OTX015 (10 μM for UM-UC-3 and SYBC1, 1 μM for T24) or DDP (2.5 μM) separately or together for 48 h. **(E)**. Western blotting assay for the levels of KLF16, MYC and apoptosis-related protein (cleaved PARP, c-PARP; cleaved Caspase3, c-Caspase3; cleaved Caspase7, c-Caspase7; cleaved Caspase9, c-Caspase9) in the indicated cells.


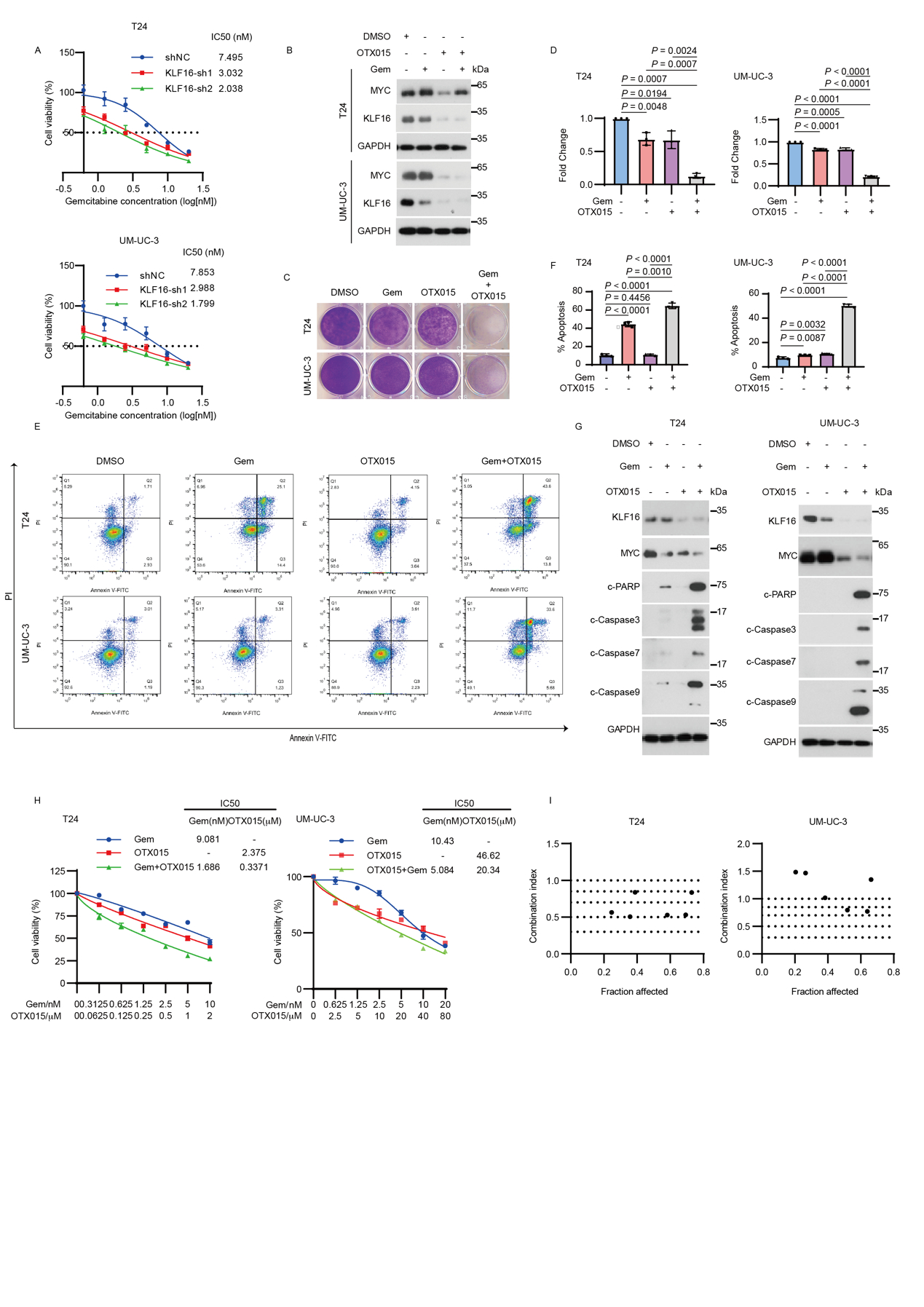


**Figure. S8. OTX015 enhances gemcitabine sensitivity in BLCA.**

**(A)**. T24 and UM-UC-3 cells expressing *KLF16-*targeted shRNAs were treated with

the indicated concentration of gemcitabine (0, 0.625 nM, 1.25 nM, 2.5 nM, 5 nM, 10 nM, 20 nM) for 60 hours, and then the cell viability was determined by MTT assays. *n* = 3 biologically independent experiments. **(B)**. T24 and UM-UC-3 cells were treated with DMSO, gemcitabine (Gem, 5 nM) or OTX015 (10 μM for UM-UC-3 and 1 μM for T24) separately or together for 72 h, the indicated proteins were determined by Western blotting. **(C-D)**. Representative images **(C)** and quantification **(D)** of colonies formed by T24 and UM-UC-3 cells used in **(B)** for about 1 week. *n* = 3 biologically independent experiments. **(E-F)**. Representative images **(E)** and quantification **(F)** of flow cytometry analysis of apoptosis in the indicated cells treated with the indicated drugs for 48 h. **(G)**. Western blotting assay for the levels of KLF16, MYC and apoptosis-related protein (cleaved PARP, c-PARP; cleaved Caspase3, c-Caspase3; cleaved Caspase7, c-Caspase7; cleaved Caspase9, c-Caspase9) in the indicated cells. **(H)**. T24 and UM-UC-3 cells were treated the indicated concentration of OTX015 or gemcitabine alone or in combination for 48 hours, and then the relative cell growth rates were determined by MTT assays. *n* = 3 biologically independent experiments. **(I)**. The Combination Index (CI) of OTX015 and gemcitabine was calculated based on results from **(G)** by using CalcuSyn**.** CI < 1, = 1, and >1 indicate synergism, additive effect, and antagonism, respectively. All error bars represent mean ± SD and *P* values in **D** and **F** were calculated using two-tailed unpaired Student’s *t*-tests.


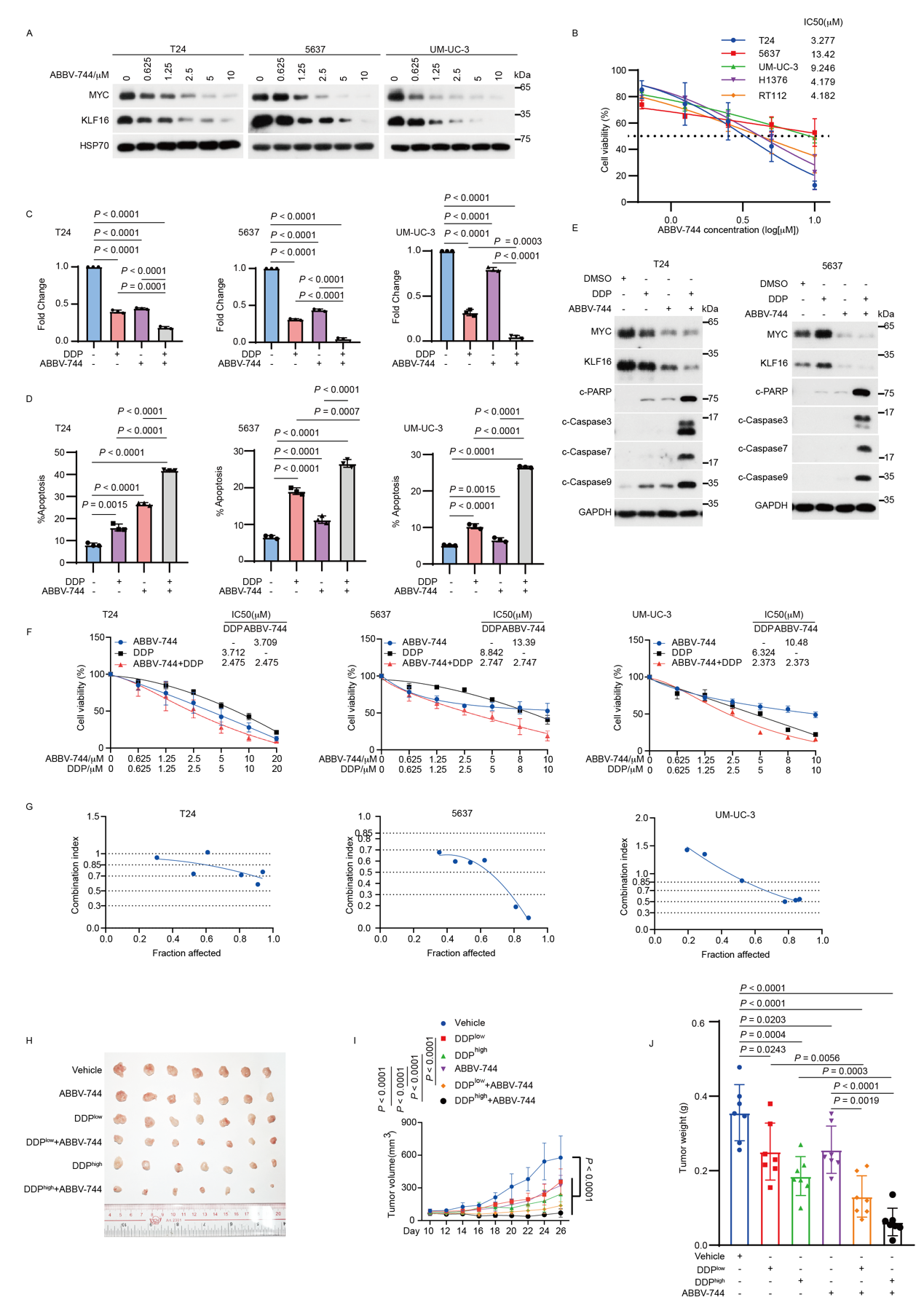


**Figure. S9.** **ABBV-744 enhances DDP sensitivity in BLCA.**

**(A).** T24, 5637 and UM-UC-3 cells were treated with increasing concentration of ABBV-744 for 48 hours and then were analyzed by Western blotting. **(B)**. The indicated bladder cancer cell lines were treated with the indicated concentration of ABBV-744 (0, 0.625 μM, 1.25 μM, 2.5 μM, 5 μM, 10 μM) for 48h, and then the relative cell growth rates were determined by MTT assay. *n* = 3 biologically independent experiments. **(C-D)**. The indicated cells were treated with DMSO, ABBV-744 (2.5 μM) or DDP (2.5 μM) separately or together, and subsequently subjected to colony formation **(C)** and flow cytometry analysis of apoptosis **(D)**. *n* = 3 biologically independent experiments. **(E)**. Western blotting assay for the levels of KLF16, MYC and apoptosis-related protein (cleaved PARP, c-PARP; cleaved Caspase3, c-Caspase3; cleaved Caspase7, c-Caspase7; cleaved Caspase9, c-Caspase9) in T24 and 5637 with the indicated drugs used in **(D)**. **(F)** BLCA cells were treated the indicated concentration of ABBV-744 or DDP alone or in combination for 48 hours, and then the relative cell growth rates were determined by MTT assays. *n* = 3 biologically independent experiments. **(G)** The Combination Index (CI) of ABBV-744 and DDP was calculated based on results from (F) by using CalcuSyn. CI < 1, = 1, and >1 indicate synergism, additive effect, and antagonism, respectively. **(H-J)**. Mice bearing T24 tumors were randomly divided into the indicated groups (*n* = 7 mice per group). DDP^low^ (3 mg kg^-1^), DDP^high^ (5 mg kg^-1^) were injected intraperitoneally twice weekly. ABBV-744 (10 mg kg^-1^) were daily given via intragastric administration. Tumor volumes **(I)** and tumor weights **(J)** were measured. All error bars represent mean ± SD and *P* values in **C**, **D**, **J** were calculated using two-tailed unpaired Student’s *t*-tests, *P* values in **I** were calculated by two-way ANOVA with Tukey’s multiple comparisons test.


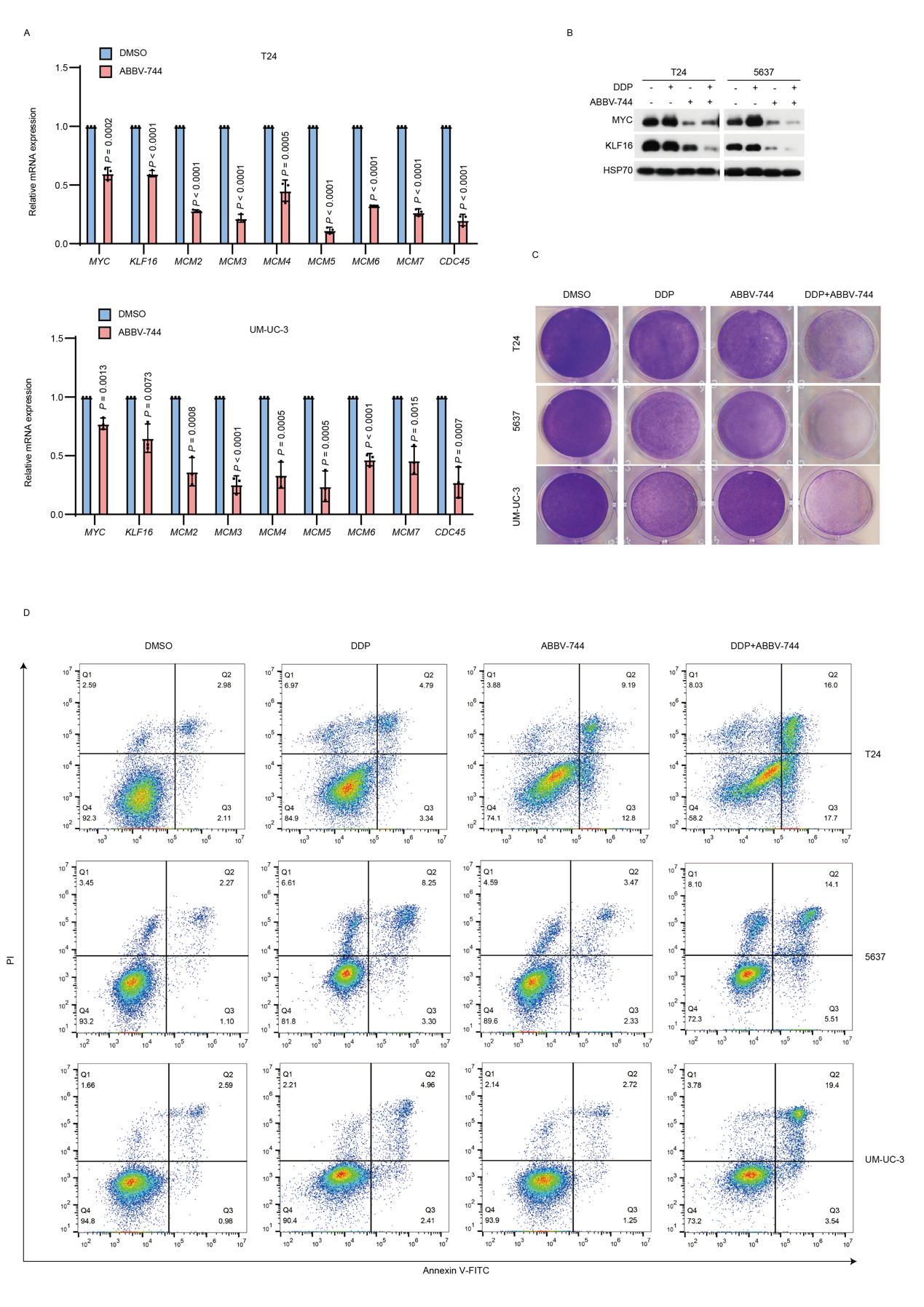


**Figure. S10. ABBV-744 enhances DDP sensitivity in BLCA.**

**(A).** T24 and UM-UC-3 cells were treated with ABBV-744 (2.5 μM) for 48 hours and then were analyzed by qRT-PCR. **(B)**. T24 and 5637 cells were treated with DMSO, ABBV-744 (2.5 μM) or DDP (2.5 μM) separately or together and subsequently subjected to Western blotting. **(C-D)**. Representative images of the indicated cells treated with DMSO, ABBV-744 (2.5 μM) or DDP (2.5 μM) separately or together and subsequently subjected to colony formation assays **(C)** and flow cytometry analysis of apoptosis **(D)**. *n* = 3 biologically independent experiments. Error bars in **(A)** represent mean ± SD and *P* values were calculated using two-tailed unpaired Student’s *t*-tests.
